# Global trend and risk factors of the disease burden for pharynx and larynx cancers between 1990 and 2019: a systematic analysis of the global burden of disease study 2019

**DOI:** 10.1186/s12889-022-14654-z

**Published:** 2022-11-28

**Authors:** Ao Huang, Xing-liang Wu, Jia Song, Yu-Ting Wang, Yin Yao, Zheng Liu, Heng Wang

**Affiliations:** 1 Department of Otolaryngology-Head and Neck Surgery, Tongji Hospital, Tongji Medical College, Huazhong University of Science and Technology, No. 1095 Jiefang Avenue, Wuhan, 430030 China; 2 Institute of Allergy and Clinical Immunology, Tongji Hospital, Tongji Medical College, Huazhong University of Science and Technology, Wuhan, China; 3 Hubei Clinical Research Center for Nasal Inflammatory Diseases, Wuhan, People’s Republic of China

**Keywords:** Pharynx and larynx cancers, Burden of disease, Public health, Risk factor, Global burden of disease study

## Abstract

**Background:**

Pharynx and larynx cancers (PLCs) are the top killer cancers in head and neck and significantly affect the quality of life of patients. A detailed study examining the disease burden and risk factors of PLCs is lacking.

**Methods:**

Data on mortality and disability-adjusted life-years (DALYs) were extracted from the Global Burden of Disease Study 2019. The estimated annual percentage change (EAPC) of the age-standardized mortality rate was calculated using a generalized linear model with a Gaussian distribution. Mortality and DALYs were stratified according to the sociodemographic index (SDI), age, gender, and risk factors. The association between the SDI and mortality rate was measured using Spearman’s correlation.

**Results:**

Between 1990 and 2019, the total number of deaths due to PLCs increased by 60.7% (95% confidence intervals: 39.32 to 66.8), from 192.38 thousand in 1990 to 309.16 thousand in 2019, and the total DALYs due to PLCs increased by 49.41% (95% confidence intervals: 30.15 to 53.27), from 5.91 million in 1990 to 8.83 million in 2019. The age-standardized mortality rate declined for larynx cancer (from 2.19 in 1990 to 1.49 in 2019) and nasopharynx cancer (1.26 to 0.86) but increased slightly for other pharynx cancer (1.25 to 1.37). The death number of PLCs was significantly higher in men aged 50 to 70 years, which accounts for 46.05% and 43.83% of the total deaths in 1990 and 2019, respectively. Low and low-middle countries had the greatest age-standardized mortality rate for larynx and other pharynx cancer, while low-middle and middle countries dominated for nasopharynx cancer. The leading risk factors for PLCs were smoking and alcohol use, which account for 37.92% and 58.84% in total DALYs rate of PLCs, and the influence of risk factors was significant in men.

**Conclusion:**

The total number of deaths and DALYs due to PLCs increased from 1990 to 2019. Countries with relatively low SDI and middle-aged and older men had the greatest burden of PLCs. Building better health care systems in relatively low SDI countries and improving strategies of smoking and alcohol control should be a priority in health policy.

**Supplementary Information:**

The online version contains supplementary material available at 10.1186/s12889-022-14654-z.

## Introduction

Pharynx and larynx cancers (PLCs) are the top killer cancers in head and neck, with 266,590 deaths reported globally in 2020, accounting for about 2.7% of all cancer deaths [1]. Until now, although there was significant improvement in the early diagnosis and treatment strategy for PLCs, a majority of patients with advanced stage PLCs are often accompanied by heavy disease burden and poor quality of life [2, 3]. Further knowledge about the burden of PLCs is urgently needed to reallocate limited health resources, which is useful for protective programs.

PLCs are associated with demographic trends, socioeconomic development, and risk exposures, including smoking, alcohol consumption, air pollution, occupational exposure, and dietary factors [4]. In the past decade, the rapid development of society and economy, drastic changes of risk factors, aging and growth of populations have occurred globally [5, 6]. As a result, the burden of PLCs has changed substantially. Previous studies of mortality and loss of health due to PLCs were based on limited data or confined to local areas [7–9]. Comprehensive estimates of the global burden of PLCs are lacking.

The Global Burden of Disease (GBD) study 2019 is an open-access database that covers 369 diseases and provides data for almost every country and territory, allowing researchers to analyze diseases in detail from many different perspectives [10]. In this study, we used data from the GBD study to explore the changing trends of mortality and disability-adjusted life-years (DALYs) of PLCs from 1990 to 2019 on a country- and region-wise level, and conducted a comprehensive analysis of risk factors, sociodemographic index (SDI), sex, and age. We hope this research can provide medical and health professionals with a deeper understanding of PLCs, thereby reducing the disease burden and contributing to health policymaking.

## Method

### Data source

Data on mortality, DALYs, age-standardized mortality rate, and age-standardized DALY rate of PLCs in 204 countries and territories from 1990 to 2019 were extracted from GBD 2019 with a specific online tool (http://ghdx.healthdata.org/gbd-results-tool) and showed a 95% uncertainty interval (UI). We used the International Classification of Diseases and Injuries-10 diagnostic codes to distinguish larynx cancer (C32), nasopharynx cancer (C11), and other pharynx cancer (C09–C10, C12–C13). The sociodemographic index (SDI) ranges from 0 (the lowest) to 1 (the highest) and divides countries and territories into five levels: low, low-middle, middle, high-middle, and high-value regions. The cut-off values used to determine quintiles for analysis were computed using country-level estimates of the SDI for the year 2019, excluding countries with populations of less than 1 million. Risk factors, including smoking, alcohol use, and occupational carcinogens, were extracted from the GBD Study 2019 and analyzed using Spearman correlation.

### Statistical analyses

The DALY or death numbers were considered to follow the formula:

$${\textrm{DV}}_{\textrm{ay},\textrm{py},\textrm{ey}}={\sum}_{i=1}^{21}\left({\textrm{a}}_{\textrm{i},\textrm{y}}\ast {\textrm{p}}_{\textrm{y}}\ast {\textrm{e}}_{\textrm{i},\textrm{y}}\right)$$

Where DV ay, py, ey represents the dependent variable (DALY or Death numbers) based on the factors of population size, age structure and risk exposure in year y. a_i,y_ represents the proportion of population for the age category i in year y, where ages under 1, above 95, and every 5 years in between were considered a category. p_y_ represents the total population in given year y; and e_i, y_ represents DALY rate or Death rate given age category i in year y.

The estimated annual percentage change (EAPC) is a widely used indicator that reflects the changing trend of the disease in a specific time interval [11]. In this study, the global age-standardized mortality rates from 1990 to 2019 and those of countries, territories, and SDI regions were estimated using the online tool and as the basic data for calculating EAPC (Supporting Table 1), which was computed underlying as follows [11]:

$$y=\upalpha +\upbeta \textbf{x}+\upvarepsilon$$

$$\textrm{EAPC}=100\ast \left(\exp \left(\upbeta \right)-1\right)$$

Where y = ln (age-standardized mortality rates), and x = calendar year. R software (version 3.5.2) was used to fit the data into a generalized linear model for calculation, and 95% confidence intervals (CI) were calculated.

The SDI of 204 countries and territories in 2019 was calculated using methods according to a previous study [12], the specific values are shown in Supporting Table 4. Spearman’s correlation was used to measure the association between SDI, risk factors and age-standardized mortality rate, the change in SDI (value of 2019 relative to 1990) and the EAPC of age-standardized mortality rate during 1990–2019 [13]. All *P* values less than 0.05 were regarded as significant.

## Result

### Disease burden and mortality estimates

According to the GBD Study 2019, DALYs due to PLCs deteriorated from 5.91 to 8.83 million between 1990 and 2019, especially for other pharynx cancer, which doubled in the same period (Supporting Table 2). The number of deaths caused by PLCs was 309.16 thousand in 2019 and 192.38 thousand in 1990. Thus, during the past 30 years, death and DALYs for PLCs increased by 60.7% (95% CI: 39.32 to 66.8%) and 49.41% (95% CI: 30.15 to 53.27), respectively. Specifically, the age-standardized mortality rate declined in larynx cancer and nasopharynx cancer but increased in other pharynx cancer. At the same time point, the gap was wider among men than among women (Supporting Table 2). Estimates of mortality rates by country are presented in Supporting Table 3. The mortality rate of PLCs in various countries around the world demonstrated a very large gap, even up to dozens of times, which will be discussed in detail later in this article.

### Other pharynx cancer

**Fig. 1:**
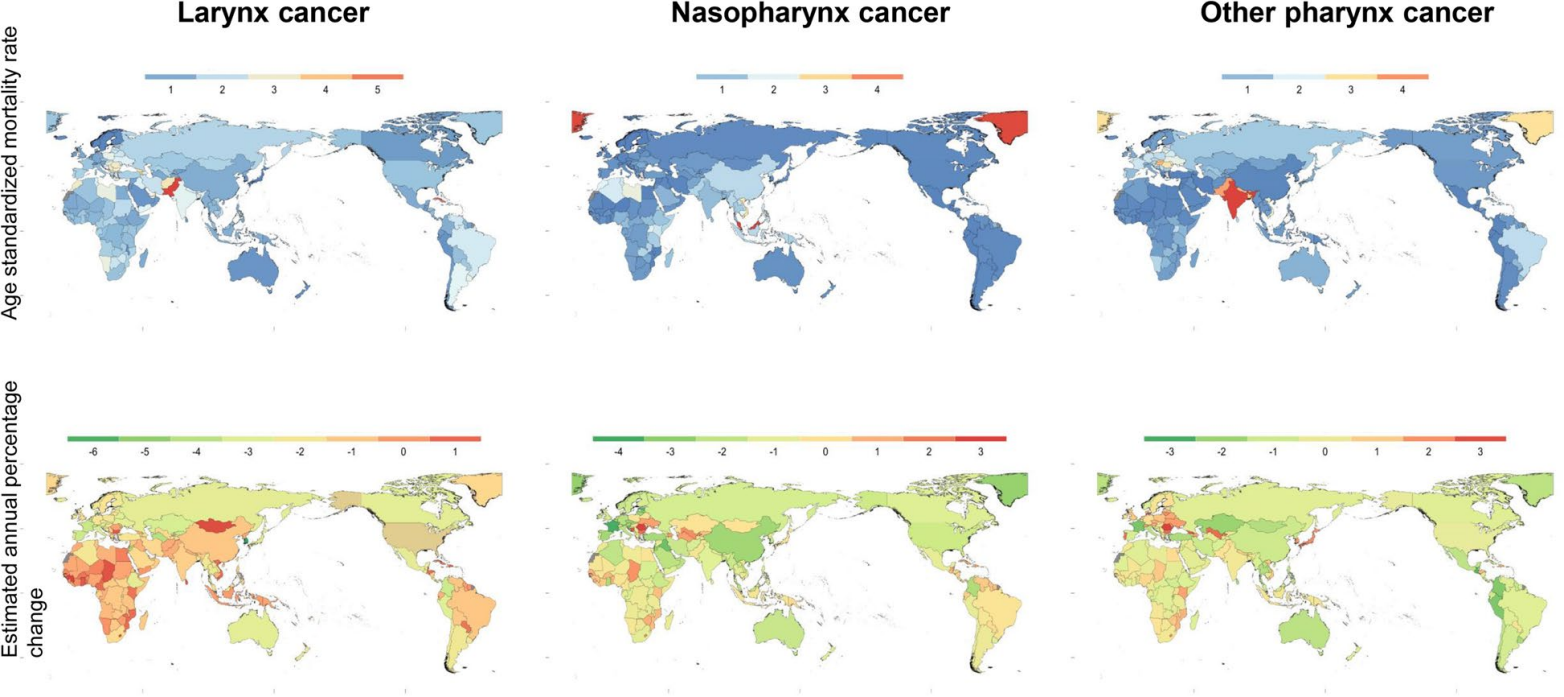
Age-standardized mortality rates of pharynx and larynx cancers for both sexes globally in 2019 (up). Estimated annual percentage change in age-standardized mortality rate per 100,000 people of pharynx and larynx cancers for both sexes globally from 1990 to 2019 (below)

In 2019, 114.2 (95% CI: 103.2 to 126.0) thousand deaths were attributed to other pharynx cancer. The age-standardized mortality rate of other pharynx cancer among countries is shown in Fig. 1. The highest rate was in India (4.89, 95% UI: 4.05 to 5.84) per 100,000 people and was much higher than that in Palestine, wherein the lowest rate was 0.19 (95% UI: 0.16 to 0.22) per 100,000 people. Countries or regions with age-standardized mortality rates above 2.0 per 100,000 people were Europe (Ukraine, Lithuania, Slovenia, Republic of Moldova, Romania, Hungary, and Slovakia), Oceania (American Samoa), Asia (Brunei Darussalam, Viet Nam, Bangladesh, Nepal, Pakistan, Bhutan, and India), North America (Greenland and Dominica), and Africa (Seychelles). Globally, the age-standardized mortality rate increased from 1.25 (95% UI: 1.17 to 1.37) per 100,000 people to 1.37 (95% UI: 1.24 to 1.51) per 100,000 people for 30 years. The average increase in the age-standardized mortality rate was 0.25% annually, with a 95% CI of 0.21 to 0.29%. The country with the most intense decrease in EAPC from 1990 to 2019 was Puerto Rico, with a 3.33% (95% CI: 3.98 to 3.68%) reduction annually. Romania occupied the other extreme, with the largest annual increase of 3.23% (95% CI: 2.97 to 3.49%) annually (Fig. 1).

### Larynx cancer

Approximately 123.3 (95% uncertainty interval: 114.9 to 132.8) thousand people died from larynx cancer in 2019, while the number was nearly 87.46 (95% UI: 83.18 to 91.55) thousand in 1990 (Supplemental Table 2). The number of deaths from larynx cancer has risen by one-third in 30 years. A global view of a wide range of age-standardized mortality rates attributable to larynx cancer in 2019 is provided in Fig. 1. The age-standardized mortality rates exceeded 5 per 100,000 people in Pakistan (5.75, 95% UI: 4.47 to 7.44), Seychelles (5.12, 95%UI: 4.34 to 6.06), and Cuba (5.03, 95% UI: 4.08 to 6.15). Mortality rates below 0.5 per 100,000 people were seen only in developed countries in Asia (Japan and Singapore), Europe (Sweden, Finland, Norway, and Iceland), and Oceania (New Zealand and Guam). From 1990 to 2019, the global age-standardized mortality rate of larynx cancer dropped by an average of 1.50% (95% CI: 1.42 to 1.57%) annually. Figure 1 shows that the country with the fastest decline in mortality rate was the Republic of Korea, with a reduction of 6.51% (95% CI: 5.90 to 7.11%). Nevertheless, the mortality rates in 35 countries increased, and the fastest increase was noted in Guinea (1.73, 95% CI: 1.53 to 1.93%).

### Nasopharynx cancer

Death number of nasopharynx cancer raised from 53.46 (95% UI: 48.87 to 57.91) thousand in 1990 to 71.61 (95% UI: 65.44 to 77.62) thousand in 2019. Figure 1 shows the global age-standardized mortality rates of nasopharynx cancer with a great difference in the regional distribution in 2019. Malaysia has a maximum age-standardized mortality rate of 4.76 (95% UI: 3.65 to 6.08) per 100,000 people in 2019. Finland had the lowest mortality rate (0.10, 95% UI: 0.09, 0.11). The mortality rate of nasopharynx cancer is generally low worldwide [14]. Other countries or territories with mortality rates of nasopharynx cancer above 2.0 were Africa (Uganda and Libya), Oceania (Solomon Islands), Asia (Taiwan [Province of China], Guam, Northern Mariana Islands, Viet Nam, and Brunei Darussalam), and North America (Greenland). The mortality rate of nasopharynx cancer showed a significant regional distribution, and the countries and regions with the highest rates were mainly concentrated in Asia. The average decrease in the global age-standardized mortality rate associated with nasopharynx cancer from 1990 to 2019 was 1.48% (95% CI: 1.39 to 1.57%) annually. The largest decrease in mortality rate was found in Singapore (4.30, 95% CI: 4.00 to 4.60%). The mortality rate with the most obvious annual increase occurred in Romania (3.02, 95% CI: 2.23 to 3.83%).

### Sex and age differences in mortality and DALYs

**Fig. 2:**
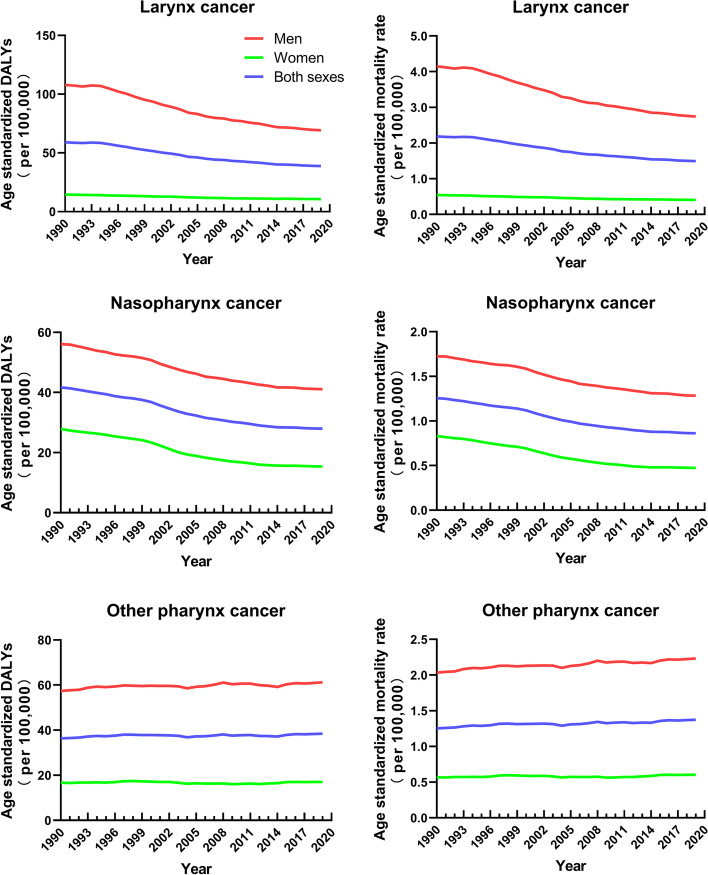
Age-standardized mortality rates (left) and disability-adjusted life-years (right) per 100,000 people of pharynx and larynx cancers among men, women, and both sexes globally from 1990 to 2019

From 1990 to 2019, the trend of the age-standardized DALY rates and age-standardized mortality rates declined for larynx cancer and nasopharynx cancer, except for other pharynx cancer, which showed a slight upward trend (Fig. 2). Both the mortality rate and the DALYs were significantly higher in men than in women. For larynx cancer, the mortality rate and DALYs among women changed in a limited manner during this period, while those of men dropped dramatically.

**Fig. 3:**
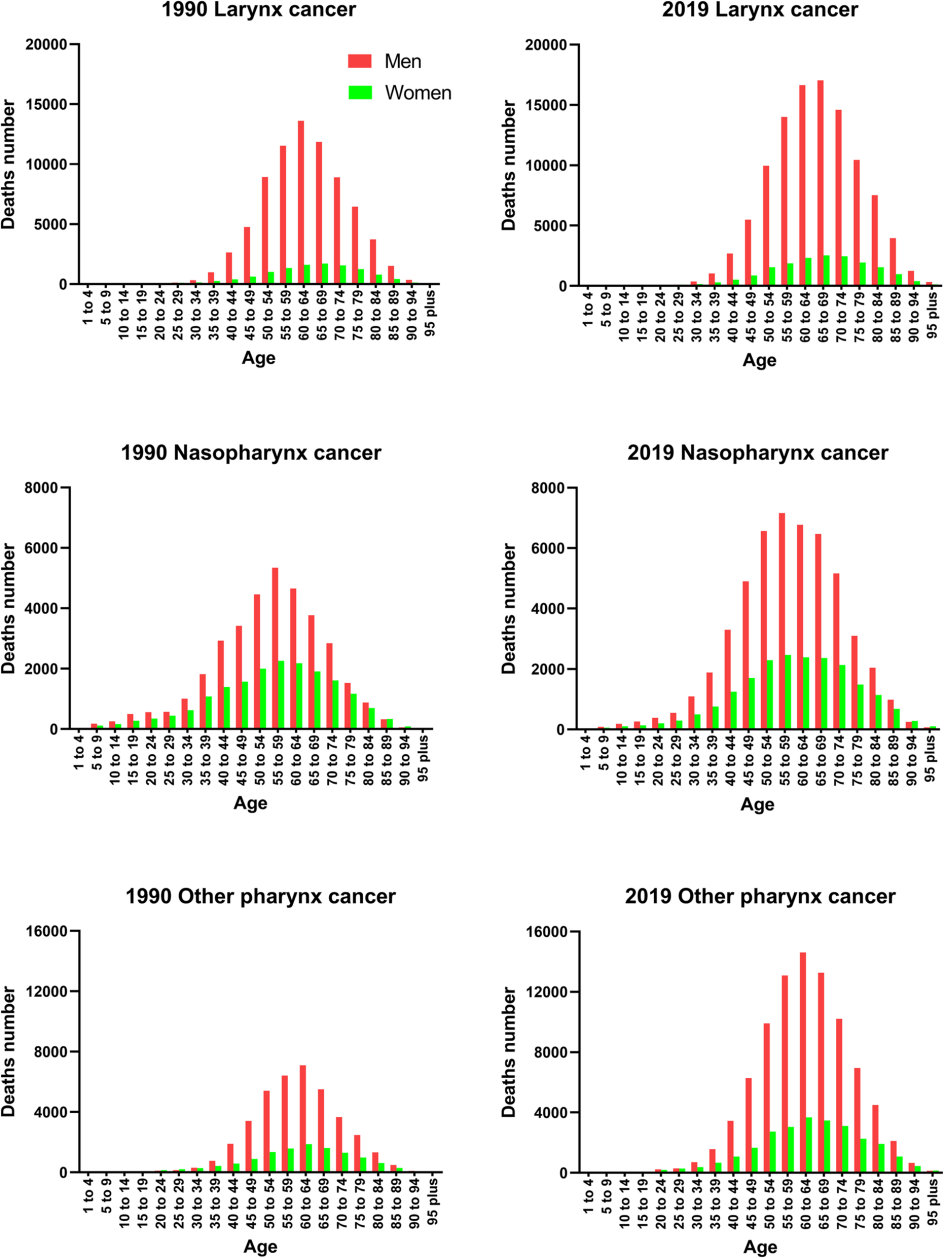
Global mortality of pharynx and larynx cancers by age among men and women in 1990 (left) and 2019 (right)

Deaths attributed globally to larynx cancer, other pharynx cancer, and nasopharynx cancer in 1990 and 2019 by age and sex are shown in Fig. 3. Deaths caused by PLCs mostly occurred among people between the ages of 50 and 70 years, and significantly affected more males, which accounts for 46.05 and 43.83% of the total deaths in 1990 and 2019, respectively.

### Correlation between the SDI and estimates of mortality and DALYs

**Fig. 4:**
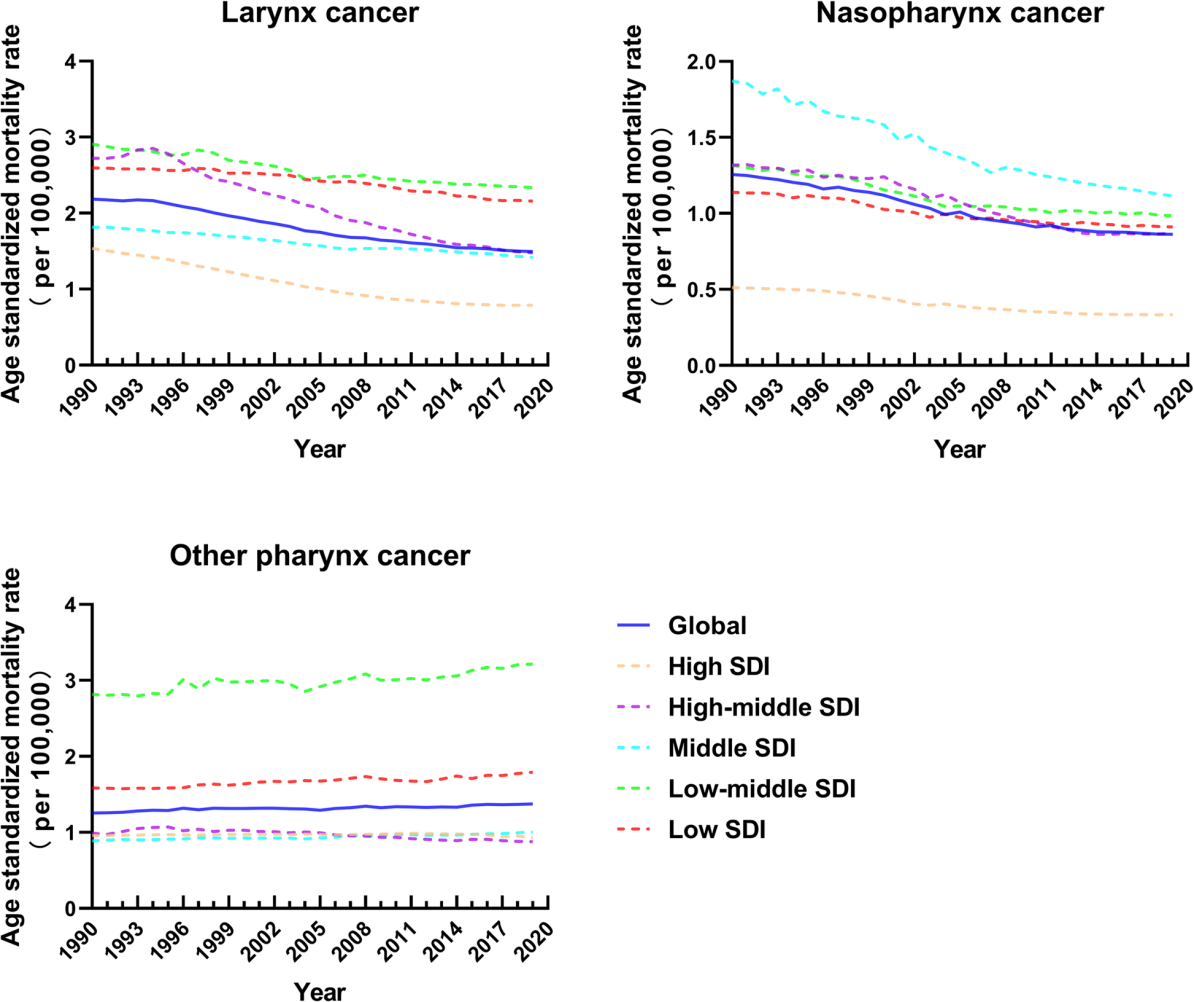
Age-standardized mortality rates per 100,000 people of larynx, nasopharynx, and other pharynx cancers grouped by sociodemographic index (SDI) quintiles from 1990 to 2019

Associations between the SDI and age-standardized mortality rates due to larynx cancer, other pharynx cancer, and nasopharynx cancer are shown in Fig. 4. From 1990 to 2019, the mortality rates of larynx cancer and nasopharynx cancer declined in all five sociodemographic regions. Low and low-middle countries had the greatest age-standardized mortality rate for larynx cancer, while low-middle and middle countries dominated for nasopharynx cancer in 2019. For other pharynx cancer, low and low-middle SDI regions showed an upward trend and a mortality rate that was much higher than that of the other three regions. The correlations between the SDI and age-standardized mortality rates of countries and territories in 2019 were explored (Supporting Fig. 1). Negative correlations were found between the mortality rates and SDI for larynx cancer (R = − 0.1511, *P* = 0.031) and nasopharynx cancer (R = − 0.268, *P* = 0.0001), while a positive correlation was identified for other pharynx cancer (R = 0.2638, *P* = 0.0001). Not surprisingly, the relationship between annual trends and age-standardized DALY rates of countries and territories between SDI was close to the result of mortality rates (Supporting Fig. 2 and Supporting Fig. 3, respectively). The change in the SDI from 1990 to 2019 globally showed no significant correlation with EAPC in the age-standardized mortality rates of nasopharynx cancer and other pharynx cancer, except for larynx cancer (R = 0. 2171, *P* = 0.0019) (Supporting Fig. 4).

### Risk factors

**Fig. 5:**
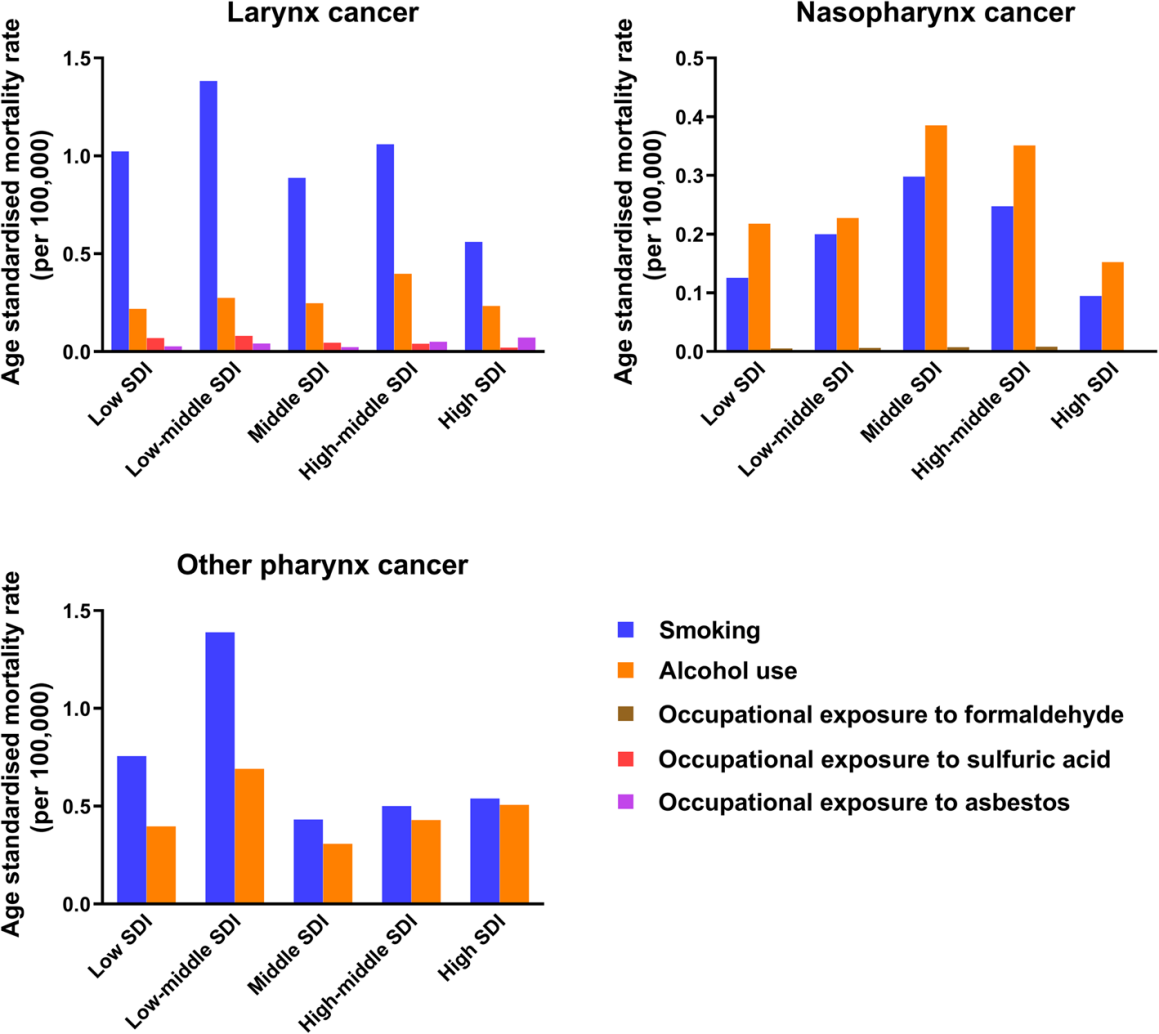
Contribution of smoking, alcohol use, and occupational carcinogens to the age-standardized mortality rates per 100,000 people of larynx cancer, nasopharynx cancer and other pharynx cancer in locations grouped by sociodemographic index quintiles in 2019

Figure 5 shows all the critical risk factors attributed to the mortality rate of PLCs in every SDI region in 2019. In all SDI quintiles, smoking and alcohol use were the two most important risk factors for death from PLCs. While smoking was primarily responsible for deaths from larynx cancer and other pharynx cancer, alcohol use was the predominant risk factor for deaths from nasopharynx cancer. In regions with low and low-middle SDI, smoking was the most common cause of death from other pharynx cancer. From 1990 to 2019, smoking and alcohol use were the two most important risk factors for mortality rates and DALYs attributable to PLCs and presented a significant downward trend except for other pharynx cancer attributed to alcohol use (Supporting Figs. 5 and 6). In 2019, approximately 2.40 million (95% UI: 2.09 to 2.73) and 1.01 million (95% UI: 0.68 to 1.27) DALYs attributable to larynx cancer were caused by smoking and alcohol use, respectively (Supporting Fig. 6). The same dominant risk factors were also observed in other pharynx cancer (1.49 million, 95% UI: 1.19 to 1.77 million), while alcohol use replaced smoking as the primary leading cause (0.96 million, 95% UI: 0.75 to 1.18) for nasopharynx cancer (Supporting Fig. 6). Smoking and alcohol use account for 37.92 and 58.84% in total DALYs rate of PLCs, respectively.

The effects of smoking and alcohol use on DALYs and mortality rates were further explored in terms of sex. Not surprisingly, both risk factors affected men far more than women (Supporting Fig. 7). For larynx cancer, DALYs attributable to occupational exposure to sulfuric acid and asbestos were relatively small (0.148 million and 0.086 million, respectively) in 2019. The effect of occupational factors on nasopharynx cancer is also shown in Supporting Fig. 6; formaldehyde contributed much less to DALYs than did alcohol use and smoking.

Deaths caused by all critical risk factors in the different SDI regions were analyzed. In 2019, the correlations between SDI and age-standardized mortality rate of larynx cancer attributable to alcohol use (R = 0.2549, *P* = 0.0002), smoking (R = 0.1547, *P* = 0.0272), and occupational asbestos exposure (R = 0.4633, *P* < 0.0001) were significantly positive, while the correlation for occupational exposure to sulfuric acid was negative (R = − 0.2322, *P* = 0.0008) (Supporting Fig. 8). Supporting Fig. 9 shows the positive correlation between SDI and age-standardized mortality rate of other pharynx cancer caused by alcohol use and smoking (R = 0.462, *P* < 0.0001 and R = 0.4111, *P* < 0.0001, respectively). Death due to nasopharynx cancer attributable to smoking (*P* = 0.6414) and alcohol use (*P* = 0.995) showed no significant relationship with SDI, while occupational exposure to formaldehyde showed a significantly negative correlation (R = − 0.4934, *P* < 0.0001) (Supporting Fig. 10).

## Discussion

The present study provided the latest comprehensive analysis of the global burden of PLCs in 204 countries during the past 30 years, and its temporal trends and attributable risk factors. Increasing trends for deaths and loss of health due to PLCs were seen. The age-standardized mortality rate declined for larynx cancer and nasopharynx cancer but increased slightly for other pharynx cancer. The disease burden of PLCs was significantly higher in middle-aged and older men. Countries with relatively low SDI carried the greatest burden of PLCs. Smoking and alcohol use were the leading causes of death due to PLCs. Building better health care systems in relatively low SDI countries and improving strategies of smoking and alcohol control should be priority in health policy.

To examine the specific factors that explain the changes in mortality and DALY rates of PLCs from 1990 to 2019, the effects of sex, age, social development, and risk exposure were examined. Differences in the mortality and DALY rates due to PLCs between males and females were seen. Male dominance has been found in PLCs’ mortality and DALY rate. It might be related to the higher prevalence of PLCs in males than females [4]. From 1990 to 2019, deaths from PLCs increased with age, especially in people aged 50 years and older. In terms of age composition, there was a mountain-like distribution of the affected population, with patients aged 50 to 70 accounting for the largest number of deaths in 2019. Therefore, death number of PLCs is likely to increase as the global population ages.

Mortality and DALYs were stratified by SDI, in order to predict mortality patterns and identify areas needing additional concern. SDI level was a key factor affecting the death rate and loss of health for high-index countries have better income conditions, which also means better health services [15]. For larynx cancer, countries with age-standardized mortality rates exceeding 5.0 per 100,000 people were Cuba (middle SDI), Pakistan (low SDI), and Seychelles (high-middle SDI), while countries with rates below 0.5 per 100,000 people were all distributed in high SDI regions. As for nasopharynx cancer, five (50%) of bottom 10 countries for mortality belonged to the high SDI region. Other pharynx cancer had the most significant mortality rates in India (middle SDI) and Bhutan (low-middle SDI). Decreasing trends in mortality rates of PLCs at the global level between 1990 and 2019 were observed in countries and regions with a high and high middle SDI. The improvement of mortality rates could be due to the unprecedented medical progress and scientific development achieved in recent years, such as advancements in surgical techniques and nonsurgical treatments, which mainly include chemotherapy, radiation therapy, immune checkpoint blockade, and oncogene-targeted therapy [16], which could be more readily available in higher level SDI countries and regions.

Smoking and alcohol use were more important risk factors for PLCs than occupational exposures and mainly affect the male population. Smoking has a dose-response relationship with head and neck cancer [17, 18]. In our study, decreasing trends of age-standardized mortality and DALY rates due to smoking of PLCs were observed. But still, smoking account for 37.92% in total age-standardized disability-adjusted life-years rate of PLCs. The prevalence of smoking has declined by 27.5% in males (men and boys) and 37.7% in females (women and girls) from 1990 to 2019, respectively [5]. Which indicates that worldwide efforts aimed at combat smoking have played a role in alleviating the disease burden of PLCs, and this effort deserve more attention.

Alcohol use increases the risk of head and neck cancer [17, 18]. The mortality and DALY rates from nasopharynx cancer and larynx cancer attributable to alcohol use presented a significant downward trend from 1990 to 2019, except for other pharynx cancer. Alcohol use account for 58.84% in total age-standardized disability-adjusted life-years rate of PLCs. A large-scale modeling study showed that the global adult alcohol use increased from 5.9 L to 6.5 L between1990 and 2017 and is forecasted to reach 7.6 L by 2030 [19]. Reducing the risk of PLCs from alcohol abuse is, therefore, important to reduce the disease burden of PLCs on a global scale. Moreover, the combination of smoking and alcohol use may have a multiplicative effect on disease burden of PLCs. Changes in at least one of these factors may have a major influence on the incidence and mortality of head and neck cancer [20]. Therefore, the most basic way of reducing the burden of PLCs is to prevent exposure to smoking and alcohol use globally [21, 22]. Improving alcohol use control strategies and persevering in current policies on smoking control are crucial for alleviating the global health burden.

### Strengths and limitations of this study

The study was based on the GBD database, which provided data on a variety of diseases in countries around the world from 1990 to 2019, allowing researchers to describe the spatial and temporal variations of diseases at different levels. This allows health policymakers to have a holistic understanding of the disease and to respond accordingly and has important implications for disease prevention in terms of etiology.

The limitations of this study are noteworthy. First, the reliability of the analysis depends on the accuracy and normalization of the data in the GBD database. Unavoidably, there may be some undetected potential bias factors in the GBD database when dealing with heterogeneous information from different databases. Moreover, data from less developed countries and regions may not be very detailed and accurate due to limited health care conditions. Second, PLCs could not be categorized into several subgroups (e.g., glottic cancer, supraglottic cancer, oral pharynx cancer, and hypopharynx cancer) according to the anatomical position because of data unavailability. A previous study reported that temporal trends differed markedly between cancers of different anatomical subsites [23]. Third, this study lacked sufficient data to determine the trends of the disease by histological subtypes. Finally, other risk factors attributable to PLCs, such as Epstein-Barr virus infection and human papilloma virus infection [24–26], were not included in the statistics; thus, the etiological discussion was limited to a certain extent.

## Conclusion

The total number of deaths and DALYs due to PLCs increased from 1990 to 2019. The age-standardized mortality rate declined for larynx cancer and nasopharynx cancer but increased slightly for other pharynx cancer. The disease burden of PLCs was significantly higher in middle-aged and older men. Countries with relatively low SDI carried the greatest burden of PLCs. Smoking and alcohol use were the leading causes of death due to PLCs. Building better health care systems in relatively low SDI countries and improving strategies of smoking and alcohol control should be priority in health policy.

## Supplementary Information

**Additional file 1: Supporting Fig. 1.** The correlation between the sociodemographic index (SDI) and age-standardized mortality rates of larynx cancer, nasopharynx cancer, and other pharynx cancer in 2019. **Supporting Fig. 2.** Age-standardized disability-adjusted life-years (DALYs) per 100,000 people of larynx cancer, nasopharynx, cancer, and other pharynx cancer grouped by sociodemographic index (SDI) quintiles from 1990 to 2019. **Supporting Fig. 3.** The correlations between the sociodemographic index (SDI) and age-standardized disability-adjusted life year rates of larynx cancer, nasopharynx cancer, and other pharynx cancer in 2019. **Supporting Fig. 4.** The correlation between the change in sociodemographic index (SDI) and estimated annual percentage change in age-standardized mortality rates of larynx cancer, nasopharynx cancer, and other pharynx cancer in 2019. **Supporting Fig. 5.** Critical risk factors contributing to age-standardized mortality rate per 100,000 people of larynx cancer, nasopharynx cancer, and other pharynx cancer between 1990 and 2019. **Supporting Fig. 6.** The age-standardized disability-adjusted life-years (DALYs) per 100,000 people of larynx, nasopharynx, and other pharynx cancer caused by all the critical risk factors between 1990 and 2019. **Supporting Fig. 7.** Age-standardized mortality rate (left, line with square symbol) and disability-adjusted life-years (DALYs) (right) per 100,000 people of pharynx and larynx cancers due to smoking and alcohol use among men and women globally from 1990 to 2019. **Supporting Fig. 8.** The correlations between sociodemographic index (SDI) and age-standardized mortality rate per 100,000 people of larynx cancer due to smoking, alcohol use, and occupational exposure to asbestos and sulfuric acid in 2019. **Supporting Fig. 9.** The correlation between sociodemographic index (SDI) and age-standardized mortality rate per 100,000 people of other pharynx cancer due to smoking and alcohol use in 2019. **Supporting Fig. 10.** The correlation between sociodemographic index (SDI) and age-standardized mortality rate per 100,000 people of nasopharynx cancer due to smoking, alcohol use, and occupational exposure to formaldehyde in 2019. **Supporting Table 1.** The EAPC of age-standardized mortality rate of larynx cancer, nasopharynx cancer, and other pharynx cancer for global, different countries and regions. **Supporting Table 2.** The global mortality, age-standardized mortality rate, and disability-adjusted life-years (DALYs) due to pharynx and larynx cancers in 1990 and 2019. **Supporting Table 3.** The age-standardized mortality rate of larynx cancer, nasopharynx cancer, and other pharynx cancer for different countries and regions in 2019. **Supporting Table 4.** The sociodemographic index (SDI) values by 204 locations in 2019.

## Data Availability

All the data were extracted from GBD 2019 with a specific online tool (http://ghdx.healthdata.org/gbd-results-tool).

## Abbreviations

PLCs: Pharynx and larynx cancers
DALYs: Disability-adjusted life-years
EAPC: The estimated annual percentage change
SDI: Sociodemographic index
GBD: Global Burden of Disease
UI: Uncertainty interval
CIs: Confidence intervals

## References

[1] Sung H, Ferlay J, Siegel RL, Laversanne M, Soerjomataram I, Jemal A, Bray F (2021). Global Cancer statistics 2020: GLOBOCAN estimates of incidence and mortality worldwide for 36 cancers in 185 countries. CA Cancer J Clin.

[2] García-León FJ, García-Estepa R, Romero-Tabares A, Gómez-Millán Borrachina J (2017). Treatment of advanced laryngeal cancer and quality of life. Systematic review. Acta Otorrinolaringol Esp.

[3] Mahalingam S, Spielmann P (2019). Quality of life outcomes following treatment of Hypopharyngeal Cancer. Adv Otorhinolaryngol.

[4] Aupérin A (2020). Epidemiology of head and neck cancers: an update. Curr Opin Oncol.

[5] Reitsma MB, Kendrick PJ, Ababneh E, Abbafati C, Abbasi-Kangevari M, Abdoli A, Abedi A, Abhilash ES, Abila DB, Aboyans V, Abu-Rmeileh NM (2021). Spatial, temporal, and demographic patterns in prevalence of smoking tobacco use and attributable disease burden in 204 countries and territories, 1990-2019: a systematic analysis from the global burden of disease study 2019. Lancet.

[6] Partridge L, Deelen J, Slagboom PE (2018). Facing up to the global challenges of ageing. Nature.

[7] Chatenoud L, Garavello W, Pagan E, Bertuccio P, Gallus S, La Vecchia C, Negri E, Bosetti C (2016). Laryngeal cancer mortality trends in European countries. Int J Cancer.

[8] Jakobsen KK, Wingstrand VL, Jensen JS, Grønhøj C, Jensen DH, Karnov K, Agander TK, Specht L, von Buchwald C (2019). Incidence and survival of hypopharyngeal cancer: a Danish nation-wide study from 1980 to 2014. Acta Oncol.

[9] Long Z, Wang W, Liu W, Wang F, Meng S, Liu J, Liu Y, Qi J, Wang L, Zhou M (2022). Trend of nasopharyngeal carcinoma mortality and years of life lost in China and its provinces from 2005 to 2020. Int J Cancer.

[10] Vos T, Lim SS, Abbafati C, Abbas KM, Abbasi M, Abbasifard M, Abbasi-Kangevari M, Abbastabar H, Abd-Allah F, Abdelalim A, Abdollahi M (2020). Global burden of 369 diseases and injuries in 204 countries and territories, 1990-2019: a systematic analysis for the global burden of disease study 2019. Lancet.

[11] Liu Z, Jiang Y, Yuan H, Fang Q, Cai N, Suo C, Jin L, Zhang T, Chen X (2019). The trends in incidence of primary liver cancer caused by specific etiologies: results from the global burden of disease study 2016 and implications for liver cancer prevention. J Hepatol.

[12] Hay SI, Abajobir AA, Abate KH, Abbafati C, Abbas KM, Abd-Allah F, Abdulkader RS, Abdulle AM, Abebo TA, Abera SF, Aboyans V (2017). Global, regional, and national disability-adjusted life-years (DALYs) for 333 diseases and injuries and healthy life expectancy (HALE) for 195 countries and territories, 1990-2016: a systematic analysis for the global burden of disease study 2016. Lancet.

[13] Li X, Cao X, Guo M, Xie M, Liu X (2020). Trends and risk factors of mortality and disability adjusted life years for chronic respiratory diseases from 1990 to 2017: systematic analysis for the global burden of disease study 2017. BMJ.

[14] Carioli G, Negri E, Kawakita D, Garavello W, La Vecchia C, Malvezzi M (2017). Global trends in nasopharyngeal cancer mortality since 1970 and predictions for 2020: focus on low-risk areas. Int J Cancer.

[15] Collaborators GBDCoD: Global (2018). Regional, and national age-sex-specific mortality for 282 causes of death in 195 countries and territories, 1980-2017: a systematic analysis for the global burden of disease study 2017. Lancet.

[16] Parmar K, Mohamed A, Vaish E, Thawani R, Cetnar J, Thein KZ (2022). Immunotherapy in head and neck squamous cell carcinoma: an updated review. Cancer Treat Res Commun.

[17] Hashibe M, Brennan P, Benhamou S, Castellsague X, Chen C, Curado MP, Dal Maso L, Daudt AW, Fabianova E, Fernandez L (2007). Alcohol drinking in never users of tobacco, cigarette smoking in never drinkers, and the risk of head and neck cancer: pooled analysis in the international head and neck Cancer epidemiology consortium. J Natl Cancer Inst.

[18] Hashim D, Genden E, Posner M, Hashibe M, Boffetta P (2019). Head and neck cancer prevention: from primary prevention to impact of clinicians on reducing burden. Ann Oncol.

[19] Manthey J, Shield KD, Rylett M, Hasan OSM, Probst C, Rehm J (2019). Global alcohol exposure between 1990 and 2017 and forecasts until 2030: a modelling study. Lancet.

[20] Hashibe M, Brennan P, Chuang SC, Boccia S, Castellsague X, Chen C, Curado MP, Dal Maso L, Daudt AW, Fabianova E (2009). Interaction between tobacco and alcohol use and the risk of head and neck cancer: pooled analysis in the international head and neck Cancer epidemiology consortium. Cancer Epidemiol Biomark Prev.

[21] Tai EW, Guy GP, Steele CB, Henley SJ, Gallaway MS, Richardson LC (2018). Cost of tobacco-related Cancer hospitalizations in the U.S., 2014. Am J Prev Med.

[22] Collaborators GBDA (2018). Alcohol use and burden for 195 countries and territories, 1990-2016: a systematic analysis for the global burden of disease study 2016. Lancet.

[23] Nahavandipour A, Jakobsen KK, Gronhoj C, Hebbelstrup Jensen D (2019). Kim Schmidt Karnov K, Klitmoller Agander T, Specht L, von Buchwald C: incidence and survival of laryngeal cancer in Denmark: a nation-wide study from 1980 to 2014. Acta Oncol.

[24] Marur S, D'Souza G, Westra WH, Forastiere AA (2010). HPV-associated head and neck cancer: a virus-related cancer epidemic. Lancet Oncol.

[25] Taberna M, Mena M, Pavon MA, Alemany L, Gillison ML, Mesia R (2017). Human papillomavirus-related oropharyngeal cancer. Ann Oncol.

[26] Chen YP, Chan ATC, Le QT, Blanchard P, Sun Y, Ma J (2019). Nasopharyngeal carcinoma. Lancet.

