# Supplementary material for: Global trend and risk factors of the disease burden for pharynx and larynx cancers between 1990 and 2019: a systematic analysis of the global burden of disease study 2019
Source: BMC Public Health. 2022 Nov 28;22:2192. doi: 10.1186/s12889-022-14654-z (PMC9703662; doi:10.1186/s12889-022-14654-z)
Supplement: Supplementary file 1 — Additional file 1: Supporting Fig. 1. The correlation between the sociodemographic index (SDI) and age-standardized mortality rates of larynx cancer, nasopharynx cancer, and other pharynx cancer in 2019. Supporting Fig. 2. Age-standardized disability-adjusted life-years (DALYs) per 100,000 people of larynx cancer, nasopharynx, cancer, and other pharynx cancer grouped by sociodemographic index (SDI) quintiles from 1990 to 2019. Supporting Fig. 3. The correlations between the sociodemographic index (SDI) and age-standardized disability-adjusted life year rates of larynx cancer, nasopharynx cancer, and other pharynx cancer in 2019. Supporting Fig. 4. The correlation between the change in sociodemographic index (SDI) and estimated annual percentage change in age-standardized mortality rates of larynx cancer, nasopharynx cancer, and other pharynx cancer in 2019. Supporting Fig. 5. Critical risk factors contributing to age-standardized mortality rate per 100,000 people of larynx cancer, nasopharynx cancer, and other pharynx cancer between 1990 and 2019. Supporting Fig. 6. The age-standardized disability-adjusted life-years (DALYs) per 100,000 people of larynx, nasopharynx, and other pharynx cancer caused by all the critical risk factors between 1990 and 2019. Supporting Fig. 7. Age-standardized mortality rate (left, line with square symbol) and disability-adjusted life-years (DALYs) (right) per 100,000 people of pharynx and larynx cancers due to smoking and alcohol use among men and women globally from 1990 to 2019. Supporting Fig. 8. The correlations between sociodemographic index (SDI) and age-standardized mortality rate per 100,000 people of larynx cancer due to smoking, alcohol use, and occupational exposure to asbestos and sulfuric acid in 2019. Supporting Fig. 9. The correlation between sociodemographic index (SDI) and age-standardized mortality rate per 100,000 people of other pharynx cancer due to smoking and alcohol use in 2019. Supporting Fig. 10. The correlatio [file 12889_2022_14654_MOESM1_ESM.docx]

**Supplementary Online Content**

**Appendix.**

**GBD Overview**

**Supporting Figure 1.** The correlation between the sociodemographic index (SDI) and age-standardized mortality rates of larynx cancer, nasopharynx cancer, and other pharynx cancer in 2019.

**Supporting Figure 2.** Age-standardized disability-adjusted life-years (DALYs) per 100,000 people of larynx cancer, nasopharynx, cancer, and other pharynx cancer grouped by socio-demographic index (SDI) quintiles from 1990 to 2019.

**Supporting Figure 3.** The correlations between the sociodemographic index (SDI) and age-standardized disability-adjusted life year rates of larynx cancer, nasopharynx cancer, and other pharynx cancer in 2019.

**Supporting Figure 4.** The correlation between the change in sociodemographic index (SDI) and estimated annual percentage change in age-standardized mortality rates of larynx cancer, nasopharynx cancer, and other pharynx cancer in 2019.

**Supporting Figure 5.** Critical risk factors contributing to age-standardized mortality rate per 100,000 people of larynx cancer, nasopharynx cancer, and other pharynx cancer between 1990 and 2019.

**Supporting Figure 6.** The age-standardized disability-adjusted life-years (DALYs) per 100,000 people of larynx, nasopharynx, and other pharynx cancer caused by all the critical risk factors between 1990 and 2019.

**Supporting Figure 7.** Age-standardized mortality rate (left, line with square symbol) and disability-adjusted life-years (DALYs) (right) per 100,000 people of pharynx and larynx cancers due to smoking and alcohol use among men and women globally from 1990 to 2019.

**Supporting Figure 8.** The correlations between sociodemographic index (SDI) and age-standardized mortality rate per 100,000 people of larynx cancer due to smoking, alcohol use, and occupational exposure to asbestos and sulfuric acid in 2019.

**Supporting Figure 9.** The correlation between sociodemographic index (SDI) and age-standardized mortality rate per 100,000 people of other pharynx cancer due to smoking and alcohol use in 2019.

**Supporting Figure 10.** The correlation between sociodemographic index (SDI) and age-standardized mortality rate per 100,000 people of nasopharynx cancer due to smoking, alcohol use, and occupational exposure to formaldehyde in 2019.

**Supporting Table 1.** The global mortality, age-standardized mortality rate, and disability-adjusted life-years (DALYs) due to pharynx and larynx cancers in 1990 and 2019.

**Supporting Table 2.** The age-standardized mortality rate of larynx cancer, nasopharynx cancer, and other pharynx cancer for different countries and regions in 2019.

**Supporting Table 3.** The sociodemographic index (SDI) values by 204 locations in 2019.

**GBD Overview**

The Global Burden of Disease (GBD) Study is the most comprehensive worldwide observational epidemiological study to date, which led by the Institute for Health Metrics and Evaluation (IHME). GBD 2019 is the latest version that provides for the first time an independent estimation of population, for each of 204 countries and territories and the globe, using a standardized, replicable approach, as well as a comprehensive update on fertility and migration. GBD is arguably the most powerful tool available to researchers to understand the changing health challenges facing people across the world. GBD 2019 incorporates major data additions and improvements, and methodological refinements. Mortality and life expectancy estimates have expanded to a total of 990 locations at the most detailed level, and new causes have been added to the fatal and non-fatal cause lists, for a total of 369 diseases and injuries, and 87 risk factors. GBD produces regular estimates of all‐cause mortality, deaths by cause, years of life lost due to premature mortality (YLLs), years lived with disability (YLDs), and disability‐adjusted life-years (DALYs) for a cause list.

**Definition of indicator**

Levels of exposure in each age-sex-location-year included in the study were estimated based on all available data sources using spatiotemporal Gaussian process regression, DisMod-MR 2.1, a Bayesian meta-regression method, or alternative methods. In this publication, estimates for the GBD cancer groups, for both sexes, and for the 5-year GBD age groups (0-5; 5-9; etc. until 95+) are presented for 204 countries or territories. We used the International Classification of Diseases and Injuries-10 diagnostic codes to distinguish larynx cancer (C32), nasopharynx cancer (C11), and other pharynx cancer (C09–C10, C12–C13). The socio-demographic index (SDI) ranges from 0 (the lowest) to 1 (the highest) and divides countries and territories into five levels: low, low-middle, middle, high-middle, and high-value regions.

**Data analysis**

The age-standardized DALY rates and death rates were decomposed based on PLCs risk factors as defined in the GBD comparative risk assessment framework which includes smoking, alcohol use, and occupational exposure (sulfuric acid, asbestos, and formaldehyde). Here we chose data from 1990 and applied the distribution of age-standardized risk factor attributable PLCs DALY and death rates to corresponding 2019 rates. For example, age-standardized rates of larynx cancer were computed based on Rate=$\sum_{q=1}^{4} R_{q,y}$. Where q represents one of the four risk factor and y represent the year and Rq,y represent the age-standardized rate for specific risk factor in given year y.

The Socio-demographic Index (SDI) is a composite indicator of development status strongly correlated with health outcomes. In short, it is the geometric mean of 0 to 1 indices of total fertility under 25 (TFU25), mean education for those aged 15 and older (EDU15+), and lag distributed income (LDI) per capita. An index score of 0 represents the minimum level of each covariate input past which selected health outcomes can get no worse, while an index score of 1 represents the maximum level of each covariate input past which selected health outcomes cease to improve. GBD 2019 SDI is calculated as it was in 2017. For GBD 2019, after calculating SDI,values were multiplied by 100 for a scale of 0 to 100. SDI was computed underlying as follows:

𝐼_𝐶𝑙𝑦_=Max ($\frac{C\mathrm{ly}-C\mathrm{low}}{Ch\mathrm{ig}h-C\mathrm{low}}$, 0.005)

I_cly_ is the index for covariate C, location l, and year y and is equal to the difference between the value of that covariate in that location‐year and the lower bound of the covariate divided by the difference between the upper and lower bounds for that covariate.

**Supporting Figure 1.** The correlation between the sociodemographic index (SDI) and age-standardized mortality rates of larynx cancer, nasopharynx cancer, and other pharynx cancer in 2019.


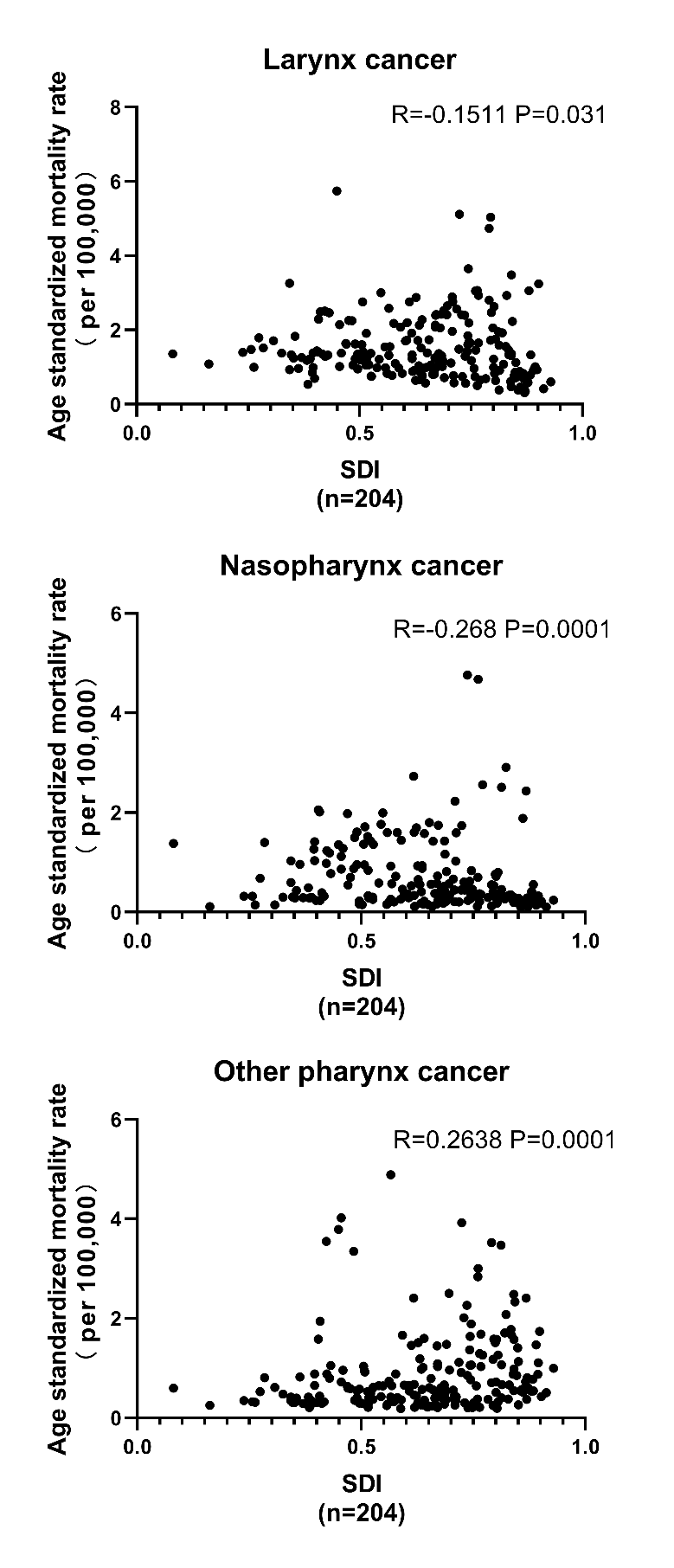


**Supporting Figure 2.** Age-standardized disability-adjusted life-years (DALYs) per 100,000 people of larynx cancer, nasopharynx, cancer, and other pharynx cancer grouped by socio-demographic index (SDI) quintiles from 1990 to 2019.


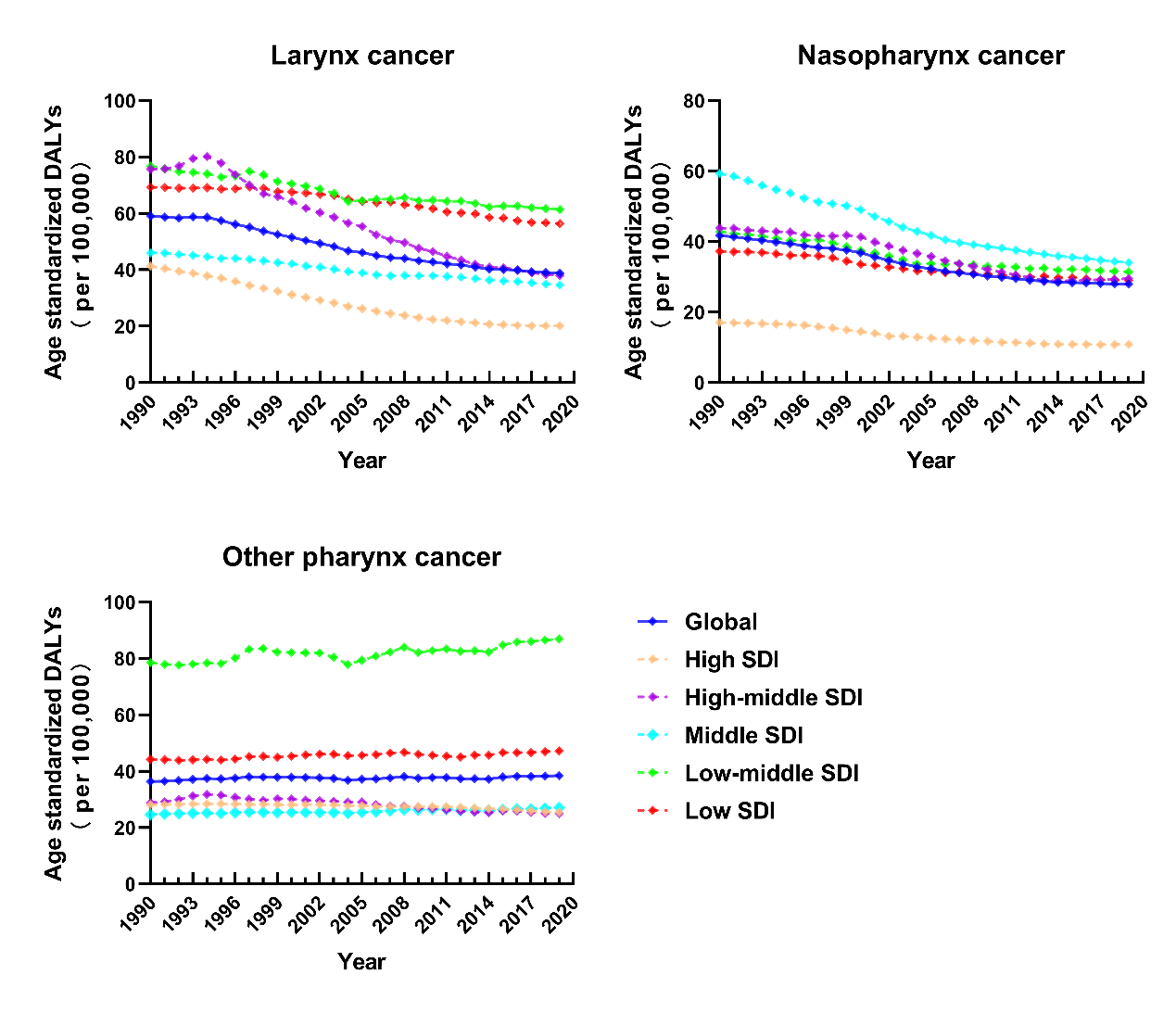


**Supporting Figure 3.** The correlations between the sociodemographic index (SDI) and age-standardized disability-adjusted life year rates of larynx cancer, nasopharynx cancer, and other pharynx cancer in 2019.


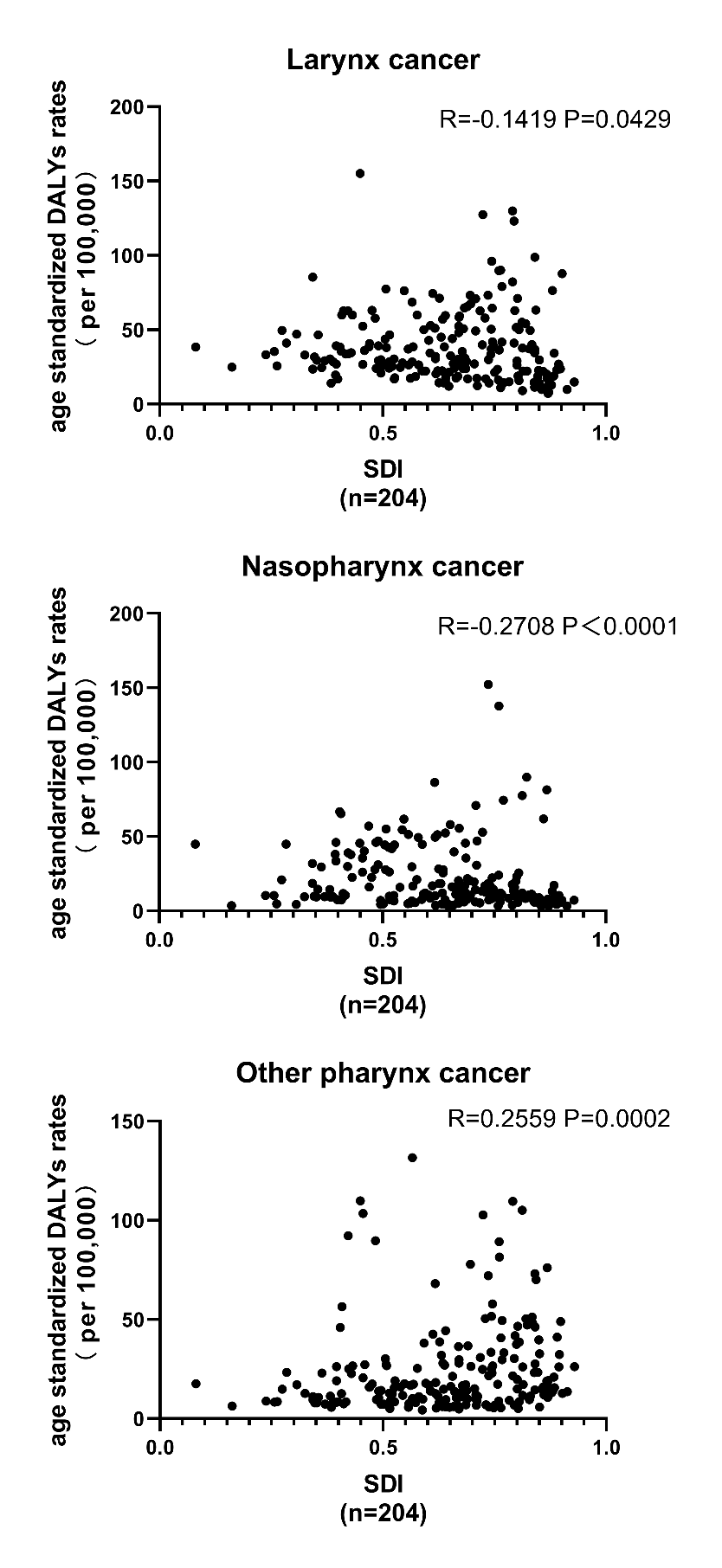


**Supporting Figure 4.** The correlation between the change in sociodemographic index (SDI) and estimated annual percentage change in age-standardized mortality rates of larynx cancer, nasopharynx cancer, and other pharynx cancer in 2019.


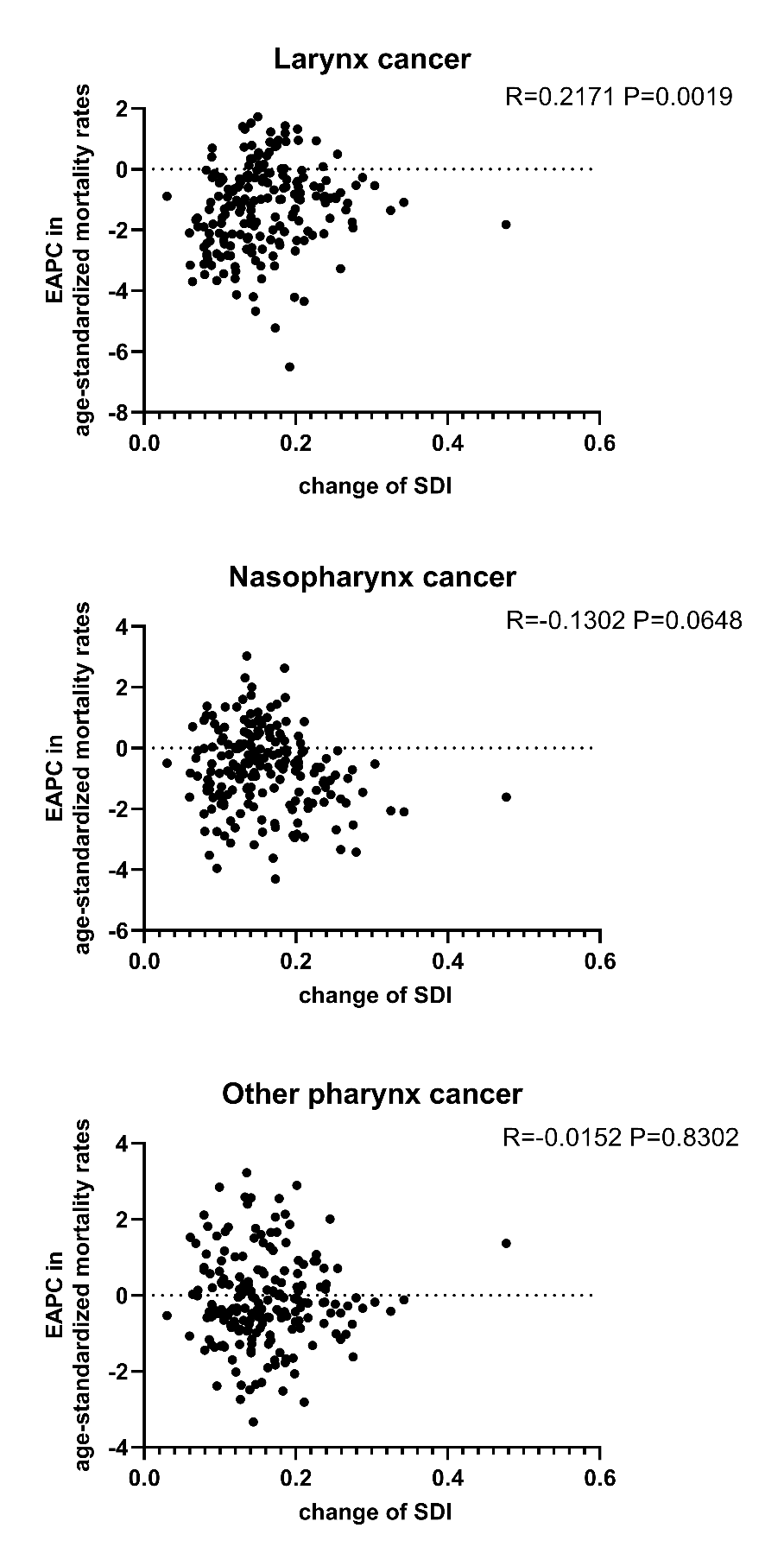


**Supporting Figure 5.** Critical risk factors contributing to age-standardized mortality rate per 100,000 people of larynx cancer, nasopharynx cancer, and other pharynx cancer between 1990 and 2019.


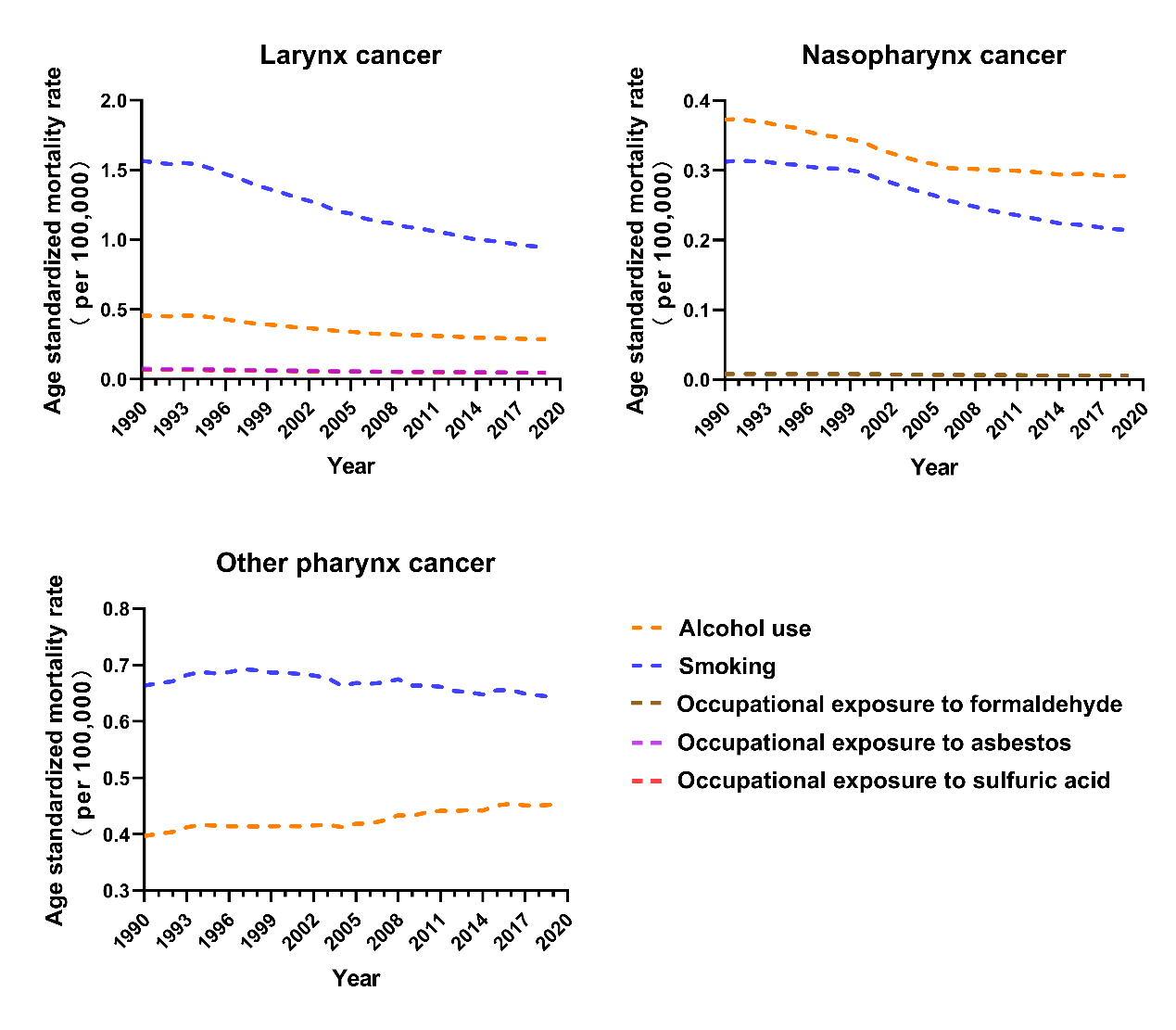


**Supporting Figure 6.** The age-standardized disability-adjusted life-years (DALYs) rate per 100,000 people of larynx, nasopharynx, and other pharynx cancer caused by all the critical risk factors between 1990 and 2019.


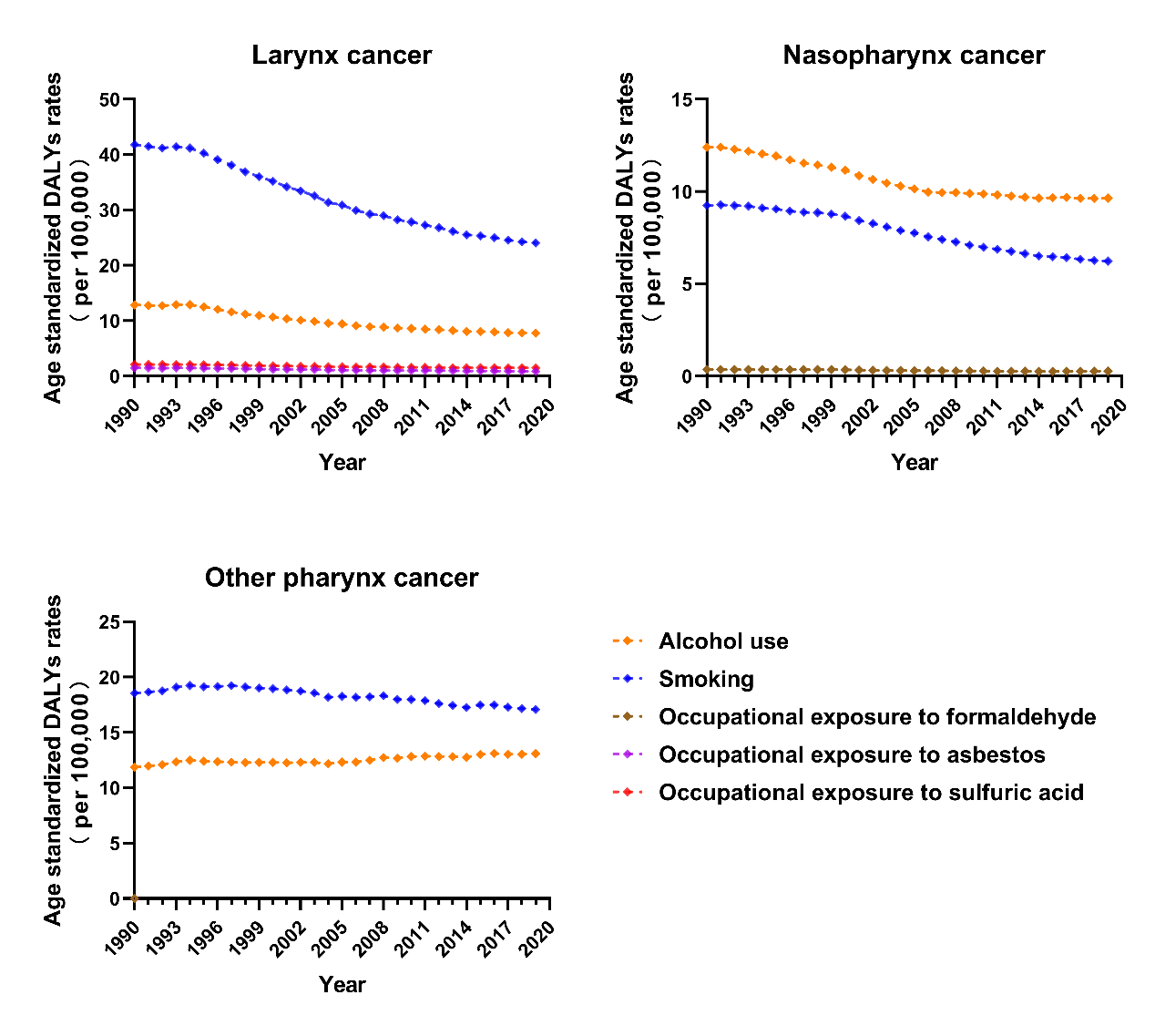


**Supporting Figure 7.** Age-standardized mortality rate (left, line with square symbol) and disability-adjusted life- years (right) per 100,000 people of pharynx and larynx cancers due to smoking and alcohol use among men and women globally from 1990 to 2019.


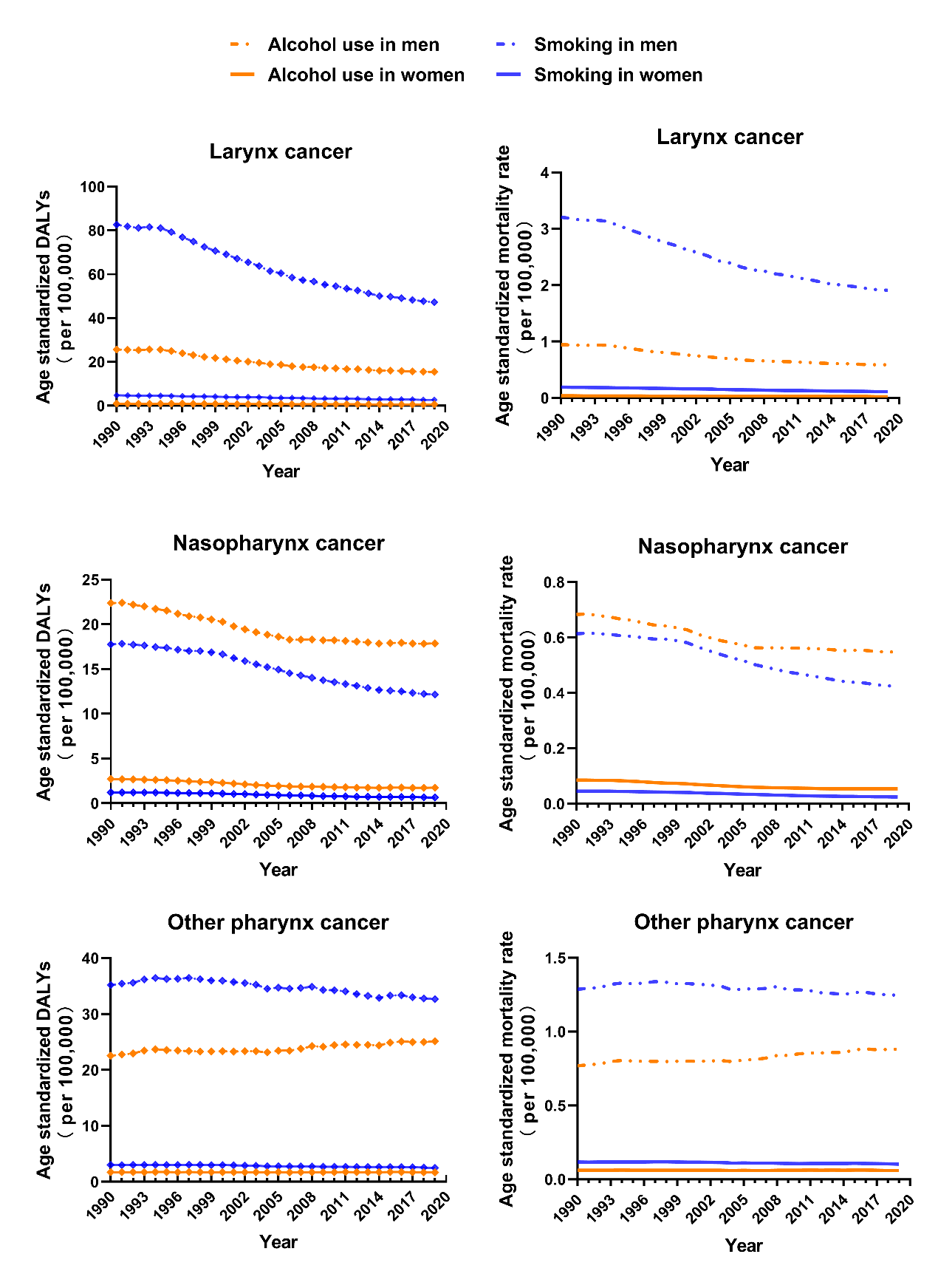


**Supporting Figure 8.** The correlations between sociodemographic index (SDI) and age-standardized mortality rate per 100,000 people of larynx cancer due to smoking, alcohol use, and occupational exposure to asbestos and sulfuric acid in 2019.


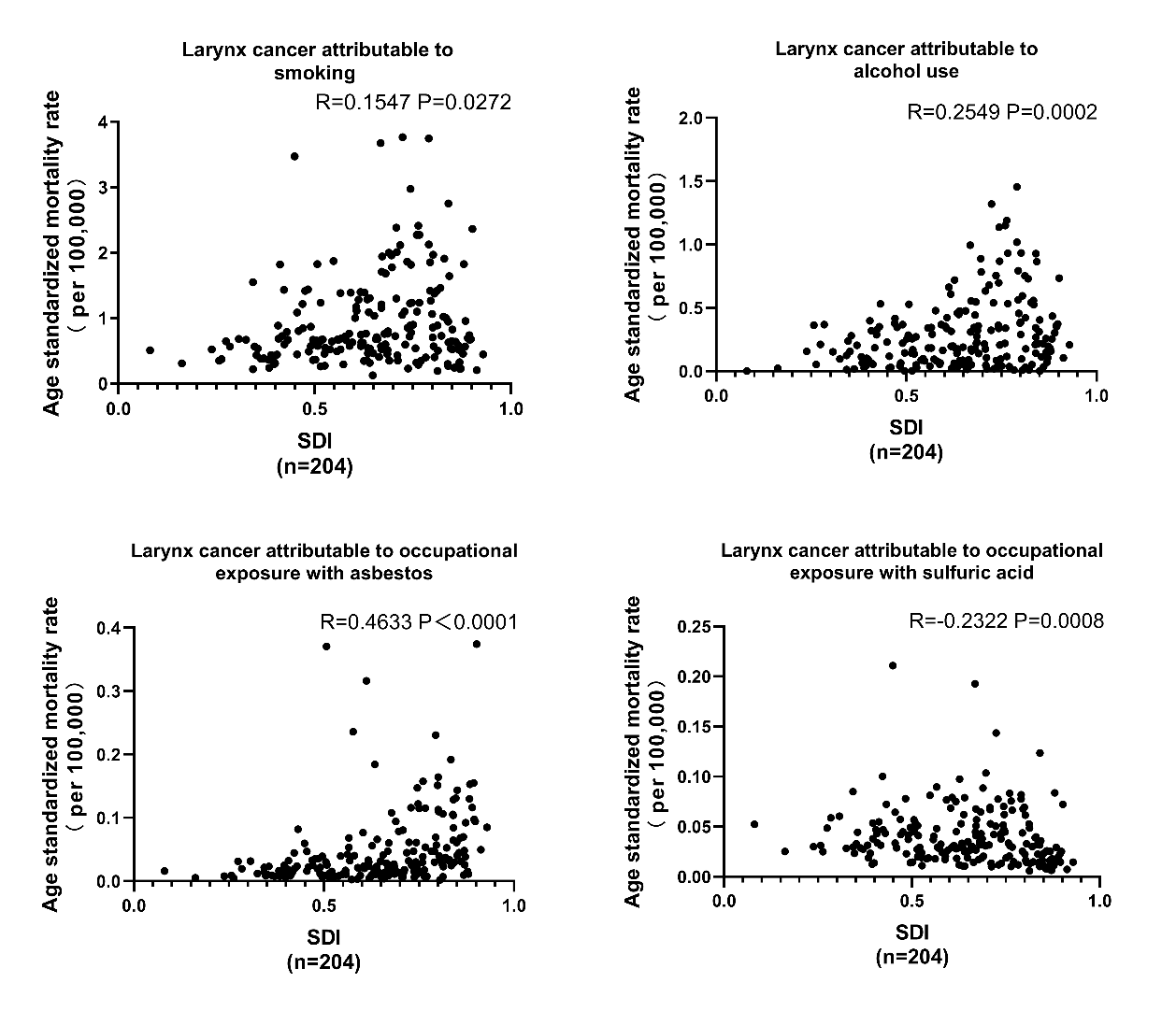


**Supporting Figure 9.** The correlation between sociodemographic index (SDI) and age-standardized mortality rate per 100,000 people of other pharynx cancer due to smoking and alcohol use in 2019.


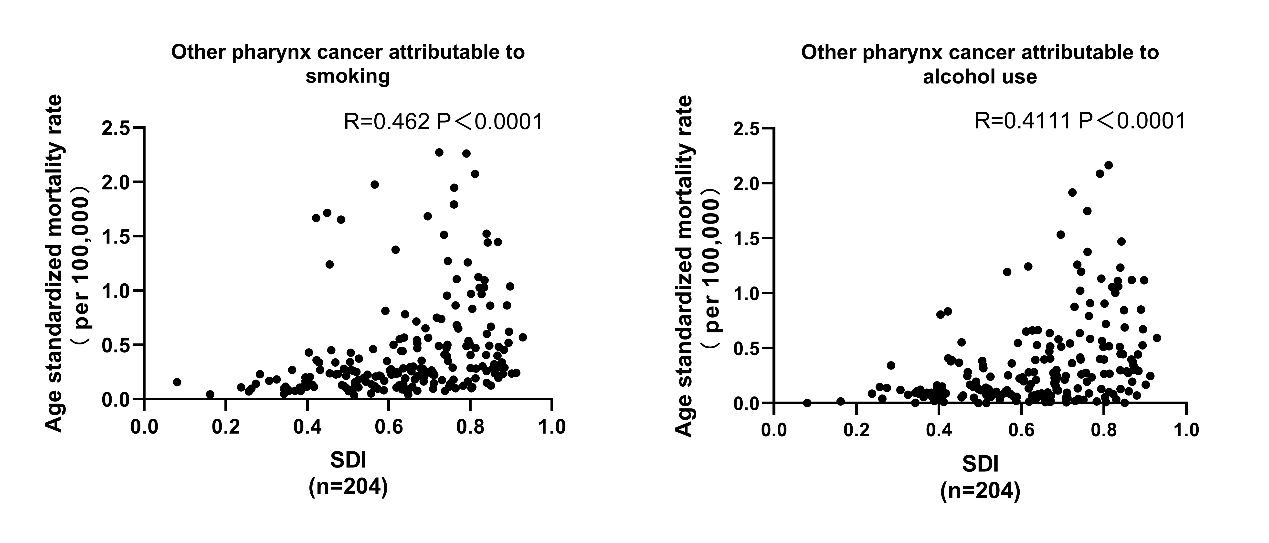


**Supporting Figure 10.** The correlation between sociodemographic index (SDI) and age-standardized mortality rate per 100,000 people of nasopharynx cancer due to smoking, alcohol use, and occupational exposure to formaldehyde in 2019.


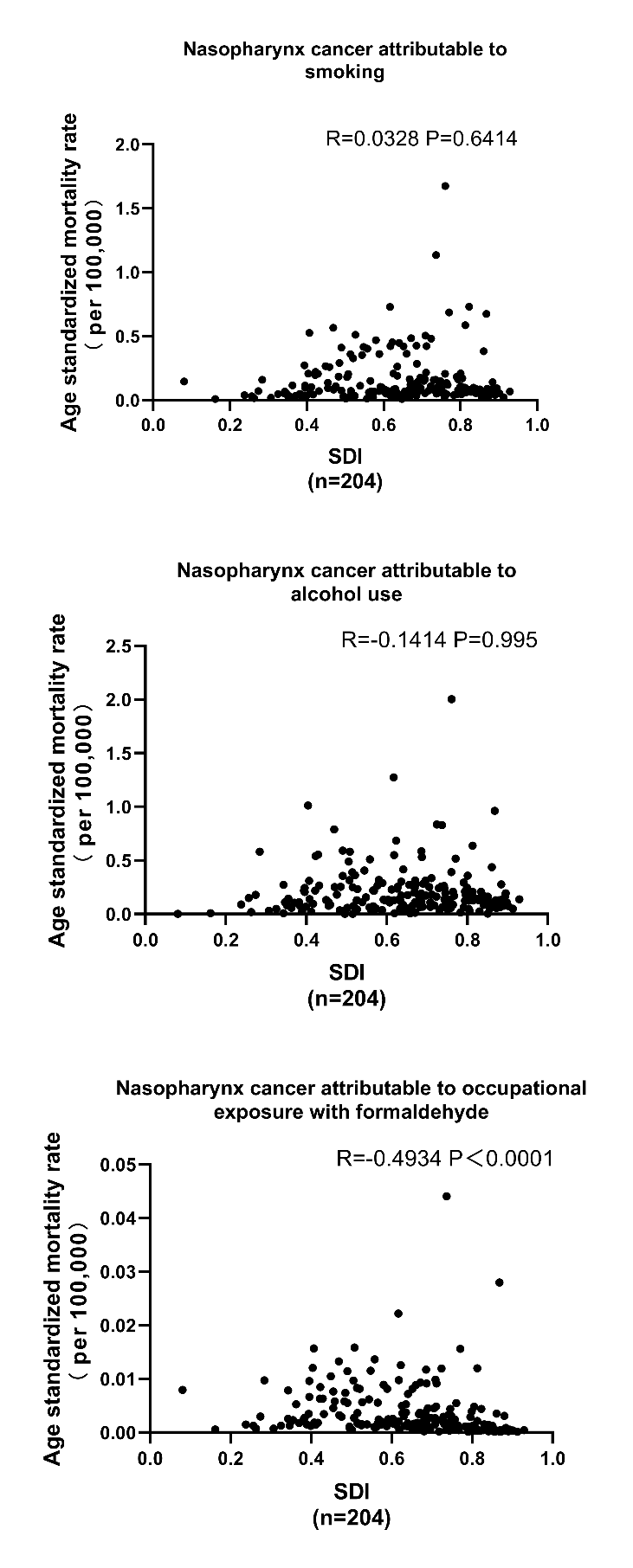


**Supporting Table 1.** The EAPC of age-standardized mortality rate of larynx cancer, nasopharynx cancer, and other pharynx cancer for global, different countries and regions.

| **location** | **Larynx cancer** | | | **Nasopharynx cancer** | | | **Other pharynx**  **cancer** | | |
| --- | --- | --- | --- | --- | --- | --- | --- | --- | --- |
|  | **EAPC** | **Lower 95%CI** | **Upper 95%CI** | **EAPC** | **Lower 95%CI** | **Upper 95%CI** | **EAPC** | **Lower 95%CI** | **Upper 95%CI** |
| **Global** | -1.50 | -1.57 | -1.42 | -1.48 | -1.57 | -1.39 | 0.25 | 0.21 | 0.29 |
| **Afghanistan** | -0.40 | -0.55 | -0.26 | -1.47 | -1.61 | -1.34 | -1.45 | -1.81 | -1.09 |
| **Albania** | -1.58 | -1.87 | -1.29 | -1.29 | -1.71 | -0.87 | -0.59 | -0.65 | -0.52 |
| **Algeria** | -2.04 | -2.27 | -1.80 | -1.98 | -2.09 | -1.87 | 1.17 | 0.79 | 1.56 |
| **American Samoa** | -2.45 | -2.68 | -2.22 | 0.69 | 0.51 | 0.87 | -1.07 | -1.12 | -1.02 |
| **Andorra** | -2.09 | -2.15 | -2.03 | -1.61 | -1.68 | -1.55 | 0.23 | 0.11 | 0.34 |
| **Angola** | -0.61 | -0.68 | -0.54 | -0.64 | -0.70 | -0.58 | -1.28 | -1.78 | -0.79 |
| **Antigua and Barbuda** | -0.11 | -0.43 | 0.20 | 0.62 | 0.44 | 0.81 | -2.74 | -3.25 | -2.23 |
| **Argentina** | -2.14 | -2.37 | -1.92 | -2.15 | -2.36 | -1.94 | -0.86 | -1.26 | -0.46 |
| **Armenia** | -2.20 | -2.37 | -2.03 | -0.58 | -0.79 | -0.36 | -1.33 | -1.44 | -1.22 |
| **Australia** | -2.89 | -3.02 | -2.76 | -1.84 | -2.04 | -1.64 | 1.56 | 1.31 | 1.82 |
| **Austria** | -2.78 | -2.87 | -2.68 | -2.75 | -2.93 | -2.57 | 1.68 | 1.23 | 2.13 |
| **Azerbaijan** | -1.48 | -1.70 | -1.26 | 1.35 | 0.85 | 1.86 | -1.31 | -1.66 | -0.97 |
| **Bahamas** | -0.33 | -0.44 | -0.23 | 0.34 | 0.25 | 0.44 | -2.07 | -2.27 | -1.86 |
| **Bahrain** | -4.22 | -4.69 | -3.74 | -2.94 | -3.42 | -2.46 | -0.20 | -0.36 | -0.05 |
| **Bangladesh** | -2.05 | -2.25 | -1.85 | -1.77 | -1.88 | -1.66 | -1.36 | -1.70 | -1.02 |
| **Barbados** | -0.13 | -0.30 | 0.03 | 0.80 | 0.54 | 1.05 | 1.61 | 1.00 | 2.21 |
| **Belarus** | -3.18 | -3.66 | -2.69 | -0.35 | -0.57 | -0.14 | 0.44 | 0.07 | 0.80 |
| **Belgium** | -3.44 | -3.56 | -3.32 | -1.89 | -2.41 | -1.37 | -0.37 | -0.58 | -0.16 |
| **Belize** | 0.88 | 0.34 | 1.43 | 1.44 | 0.91 | 1.97 | -0.04 | -0.09 | 0.02 |
| **Benin** | 0.35 | 0.22 | 0.48 | 0.81 | 0.72 | 0.90 | -2.36 | -2.84 | -1.88 |
| **Bermuda** | -1.28 | -1.42 | -1.13 | -0.68 | -0.73 | -0.63 | 0.90 | 0.83 | 0.98 |
| **Bhutan** | -0.88 | -0.94 | -0.82 | -1.39 | -1.52 | -1.26 | -0.55 | -0.67 | -0.42 |
| **Bolivia (Plurinational State of)** | -0.99 | -1.17 | -0.80 | -0.34 | -0.38 | -0.29 | -0.43 | -0.57 | -0.29 |
| **Bosnia and Herzegovina** | -2.05 | -2.32 | -1.79 | 2.63 | 2.00 | 3.27 | 0.11 | -0.40 | 0.63 |
| **Botswana** | -0.97 | -1.53 | -0.41 | -0.47 | -0.81 | -0.14 | -0.56 | -0.72 | -0.39 |
| **Brazil** | -0.96 | -1.04 | -0.89 | -0.03 | -0.41 | 0.35 | 1.76 | 1.35 | 2.17 |
| **Brunei Darussalam** | -4.67 | -4.98 | -4.37 | -0.26 | -0.51 | -0.02 | 2.59 | 2.10 | 3.08 |
| **Bulgaria** | 1.32 | 0.94 | 1.71 | 2.32 | 2.05 | 2.58 | 0.19 | 0.04 | 0.35 |
| **Burkina Faso** | 0.73 | 0.59 | 0.88 | 0.93 | 0.83 | 1.03 | -1.17 | -1.30 | -1.03 |
| **Burundi** | -2.11 | -2.28 | -1.94 | -1.36 | -1.50 | -1.23 | 2.68 | 1.98 | 3.39 |
| **Cabo Verde** | -1.41 | -1.96 | -0.87 | 2.90 | 2.03 | 3.77 | -0.13 | -0.29 | 0.04 |
| **Cambodia** | -0.58 | -0.80 | -0.36 | -0.61 | -0.69 | -0.54 | 0.00 | -0.05 | 0.05 |
| **Cameroon** | 0.95 | 0.74 | 1.16 | -0.17 | -0.30 | -0.05 | -0.58 | -0.80 | -0.36 |
| **Canada** | -2.85 | -3.02 | -2.69 | -1.27 | -1.46 | -1.08 | -0.57 | -0.61 | -0.53 |
| **Central African Republic** | -1.08 | -1.11 | -1.05 | -0.77 | -0.82 | -0.72 | 1.03 | 0.94 | 1.12 |
| **Chad** | 1.40 | 1.19 | 1.62 | 1.61 | 1.36 | 1.85 | -1.18 | -1.36 | -1.01 |
| **Chile** | -2.32 | -2.46 | -2.18 | -0.86 | -1.14 | -0.58 | -1.01 | -1.18 | -0.83 |
| **China** | -0.96 | -1.07 | -0.86 | -2.69 | -2.81 | -2.57 | -2.29 | -2.52 | -2.07 |
| **Colombia** | -3.60 | -3.85 | -3.36 | -2.36 | -2.53 | -2.20 | -0.59 | -0.76 | -0.41 |
| **Comoros** | -0.99 | -1.17 | -0.81 | -0.62 | -0.83 | -0.41 | -0.66 | -0.88 | -0.45 |
| **Congo** | -1.70 | -1.92 | -1.48 | -1.44 | -1.61 | -1.28 | -0.66 | -0.85 | -0.48 |
| **Cook Islands** | -1.78 | -2.04 | -1.52 | -1.56 | -1.82 | -1.30 | -0.98 | -1.24 | -0.73 |
| **Costa Rica** | -1.74 | -1.93 | -1.55 | -0.43 | -0.62 | -0.23 | -0.78 | -0.95 | -0.60 |
| **Croatia** | -2.52 | -2.66 | -2.38 | -2.39 | -2.58 | -2.21 | -0.54 | -1.13 | 0.05 |
| **Cuba** | 0.69 | 0.60 | 0.79 | 1.07 | 0.96 | 1.17 | 0.04 | -0.14 | 0.23 |
| **Cyprus** | -1.86 | -2.05 | -1.67 | -0.51 | -0.71 | -0.32 | 1.44 | 1.19 | 1.70 |
| **Czechia** | -2.30 | -2.37 | -2.23 | -2.83 | -2.98 | -2.67 | -0.55 | -0.66 | -0.45 |
| **Côte d'Ivoire** | 0.15 | -0.01 | 0.30 | 0.50 | 0.33 | 0.67 | -0.90 | -0.97 | -0.83 |
| **Democratic People's Republic of Korea** | -0.43 | -0.49 | -0.36 | -0.82 | -0.88 | -0.75 | -0.43 | -0.65 | -0.20 |
| **Democratic Republic of the Congo** | -1.02 | -1.12 | -0.93 | -0.62 | -0.68 | -0.57 | 1.81 | 1.29 | 2.34 |
| **Denmark** | -2.97 | -3.06 | -2.88 | -1.04 | -1.24 | -0.84 | -0.05 | -0.11 | 0.00 |
| **Djibouti** | -0.62 | -0.74 | -0.51 | -0.57 | -0.68 | -0.47 | -0.56 | -0.92 | -0.19 |
| **Dominica** | 0.45 | 0.37 | 0.52 | 1.18 | 1.08 | 1.29 | 1.65 | 1.47 | 1.84 |
| **Dominican Republic** | 1.23 | 0.95 | 1.51 | 1.35 | 0.99 | 1.71 | -0.93 | -1.20 | -0.66 |
| **Ecuador** | -0.60 | -0.85 | -0.35 | 0.47 | 0.25 | 0.70 | 0.71 | 0.45 | 0.98 |
| **Egypt** | 0.49 | 0.28 | 0.71 | -0.09 | -0.35 | 0.17 | -2.52 | -2.93 | -2.11 |
| **El Salvador** | -0.19 | -0.44 | 0.05 | -0.68 | -1.06 | -0.31 | 1.37 | 1.21 | 1.54 |
| **Equatorial Guinea** | -1.82 | -2.23 | -1.41 | -1.61 | -1.93 | -1.29 | -0.08 | -0.34 | 0.18 |
| **Eritrea** | -0.82 | -0.97 | -0.67 | -0.49 | -0.61 | -0.37 | 0.11 | -0.10 | 0.33 |
| **Estonia** | -2.86 | -3.13 | -2.59 | -3.62 | -3.97 | -3.27 | 0.65 | 0.19 | 1.11 |
| **Eswatini** | 0.02 | -0.43 | 0.48 | 0.24 | -0.24 | 0.72 | -0.69 | -0.90 | -0.48 |
| **Ethiopia** | -2.68 | -2.81 | -2.55 | -1.74 | -1.88 | -1.60 | 0.36 | 0.14 | 0.59 |
| **Fiji** | -0.19 | -0.35 | -0.03 | -0.51 | -0.84 | -0.18 | 0.64 | 0.41 | 0.87 |
| **Finland** | -2.11 | -2.29 | -1.93 | -1.50 | -1.60 | -1.40 | -2.38 | -2.54 | -2.22 |
| **France** | -3.66 | -4.03 | -3.29 | -3.95 | -4.14 | -3.76 | -0.28 | -0.33 | -0.23 |
| **Gabon** | -1.11 | -1.16 | -1.06 | -1.00 | -1.06 | -0.93 | 0.34 | 0.17 | 0.50 |
| **Gambia** | 0.01 | -0.15 | 0.18 | 0.35 | 0.16 | 0.54 | 2.85 | 2.03 | 3.67 |
| **Georgia** | -0.44 | -0.82 | -0.07 | -0.83 | -1.18 | -0.48 | 0.67 | 0.45 | 0.89 |
| **Germany** | -1.90 | -1.96 | -1.84 | -2.16 | -2.39 | -1.94 | -0.14 | -0.27 | -0.02 |
| **Ghana** | 1.32 | 1.09 | 1.56 | -2.46 | -2.95 | -1.98 | 0.28 | 0.15 | 0.42 |
| **Greece** | -1.21 | -1.28 | -1.14 | -0.21 | -0.40 | -0.02 | -1.36 | -1.54 | -1.18 |
| **Greenland** | -1.30 | -1.48 | -1.12 | -2.88 | -3.09 | -2.68 | -0.86 | -1.29 | -0.43 |
| **Grenada** | -0.78 | -1.17 | -0.38 | 0.16 | -0.15 | 0.48 | 1.02 | 0.45 | 1.59 |
| **Guam** | -3.21 | -3.56 | -2.85 | 0.03 | -0.27 | 0.32 | -2.81 | -3.18 | -2.44 |
| **Guatemala** | -2.35 | -2.60 | -2.09 | 0.87 | -0.23 | 1.98 | 0.68 | 0.61 | 0.74 |
| **Guinea** | 1.73 | 1.53 | 1.93 | 0.57 | 0.48 | 0.66 | -0.36 | -0.42 | -0.30 |
| **Guinea-Bissau** | 0.06 | -0.12 | 0.24 | 0.79 | 0.66 | 0.93 | -1.05 | -1.34 | -0.75 |
| **Guyana** | -0.12 | -0.22 | -0.03 | 0.65 | 0.45 | 0.86 | -0.94 | -1.11 | -0.76 |
| **Haiti** | -0.55 | -0.69 | -0.41 | 0.05 | -0.01 | 0.11 | 1.28 | 1.11 | 1.45 |
| **Honduras** | 0.90 | 0.69 | 1.10 | 0.35 | 0.20 | 0.50 | 0.29 | -0.66 | 1.26 |
| **Hungary** | -1.79 | -2.04 | -1.55 | 0.14 | -0.36 | 0.64 | -0.65 | -0.73 | -0.57 |
| **Iceland** | -2.34 | -2.56 | -2.13 | -1.81 | -2.04 | -1.58 | 0.16 | 0.07 | 0.26 |
| **India** | -1.11 | -1.23 | -0.99 | -1.29 | -1.53 | -1.05 | 0.26 | 0.19 | 0.33 |
| **Indonesia** | -0.04 | -0.12 | 0.04 | -0.14 | -0.23 | -0.06 | -1.02 | -1.15 | -0.89 |
| **Iran (Islamic Republic of)** | -1.34 | -1.53 | -1.14 | -1.80 | -1.93 | -1.67 | -0.06 | -0.18 | 0.06 |
| **Iraq** | -0.53 | -0.63 | -0.43 | -3.42 | -3.92 | -2.91 | 0.11 | -0.05 | 0.27 |
| **Ireland** | -2.24 | -2.37 | -2.11 | -1.84 | -2.17 | -1.51 | -0.43 | -0.51 | -0.34 |
| **Israel** | -1.32 | -1.59 | -1.05 | -3.52 | -3.91 | -3.13 | -1.29 | -1.36 | -1.23 |
| **Italy** | -3.16 | -3.29 | -3.04 | -2.01 | -2.13 | -1.88 | -0.48 | -0.98 | 0.02 |
| **Jamaica** | 0.78 | 0.34 | 1.23 | 2.01 | 1.57 | 2.45 | 2.12 | 1.75 | 2.48 |
| **Japan** | -2.57 | -2.65 | -2.49 | -0.02 | -0.38 | 0.35 | -0.19 | -0.33 | -0.05 |
| **Jordan** | -4.35 | -4.98 | -3.71 | -2.93 | -3.22 | -2.64 | -2.02 | -2.29 | -1.75 |
| **Kazakhstan** | -3.36 | -3.57 | -3.15 | -0.02 | -0.30 | 0.25 | 1.66 | 1.45 | 1.87 |
| **Kenya** | 0.78 | 0.50 | 1.07 | 0.75 | 0.57 | 0.94 | -0.37 | -0.47 | -0.27 |
| **Kiribati** | -0.39 | -0.44 | -0.34 | -0.25 | -0.37 | -0.14 | -1.65 | -2.00 | -1.30 |
| **Kuwait** | -1.47 | -1.83 | -1.09 | -2.87 | -3.24 | -2.51 | 0.03 | -0.26 | 0.33 |
| **Kyrgyzstan** | -3.70 | -4.10 | -3.29 | 0.71 | 0.44 | 0.97 | -1.32 | -1.51 | -1.12 |
| **Lao People's Democratic Republic** | -2.17 | -2.34 | -2.00 | -1.81 | -1.91 | -1.71 | 1.51 | 1.13 | 1.89 |
| **Latvia** | -1.86 | -2.20 | -1.51 | -3.18 | -3.52 | -2.84 | -0.46 | -0.59 | -0.34 |
| **Lebanon** | -0.95 | -1.15 | -0.75 | -1.53 | -1.70 | -1.36 | 2.13 | 1.85 | 2.42 |
| **Lesotho** | 1.43 | 1.14 | 1.71 | 1.66 | 1.37 | 1.96 | -0.39 | -0.66 | -0.11 |
| **Liberia** | -0.04 | -0.23 | 0.15 | 0.41 | 0.26 | 0.56 | -0.18 | -0.28 | -0.07 |
| **Libya** | -0.54 | -0.71 | -0.36 | -0.52 | -0.65 | -0.40 | 2.06 | 1.83 | 2.30 |
| **Lithuania** | -1.57 | -1.91 | -1.23 | -2.61 | -2.80 | -2.41 | -1.45 | -1.58 | -1.31 |
| **Luxembourg** | -3.46 | -3.54 | -3.38 | -2.74 | -2.82 | -2.66 | -0.66 | -0.75 | -0.56 |
| **Madagascar** | -1.05 | -1.16 | -0.93 | -0.85 | -0.93 | -0.78 | 0.07 | -0.01 | 0.14 |
| **Malawi** | -0.68 | -0.87 | -0.49 | -1.31 | -1.53 | -1.09 | -0.89 | -1.14 | -0.64 |
| **Malaysia** | -1.57 | -1.88 | -1.25 | -2.02 | -2.40 | -1.64 | -0.46 | -0.75 | -0.18 |
| **Maldives** | -3.27 | -3.52 | -3.03 | -3.34 | -3.54 | -3.14 | 0.28 | 0.15 | 0.41 |
| **Mali** | -0.30 | -0.41 | -0.18 | -0.22 | -0.37 | -0.08 | -0.94 | -1.13 | -0.74 |
| **Malta** | -2.63 | -2.73 | -2.52 | -1.33 | -1.53 | -1.14 | -0.07 | -0.15 | 0.00 |
| **Marshall Islands** | -0.28 | -0.42 | -0.15 | -0.12 | -0.22 | -0.01 | -0.54 | -0.70 | -0.39 |
| **Mauritania** | -0.34 | -0.58 | -0.10 | -0.14 | -0.29 | 0.00 | 2.55 | 2.07 | 3.03 |
| **Mauritius** | -2.41 | -2.60 | -2.22 | -0.55 | -0.92 | -0.18 | -1.27 | -1.41 | -1.14 |
| **Mexico** | -2.76 | -2.91 | -2.60 | -0.93 | -1.02 | -0.84 | -0.33 | -0.46 | -0.20 |
| **Micronesia (Federated States of)** | -0.98 | -1.09 | -0.87 | -0.48 | -0.63 | -0.34 | 1.37 | 1.02 | 1.72 |
| **Monaco** | -1.66 | -1.83 | -1.48 | -0.33 | -0.42 | -0.23 | -1.51 | -1.78 | -1.24 |
| **Mongolia** | 1.52 | 1.16 | 1.88 | 0.07 | -0.16 | 0.30 | 0.20 | 0.13 | 0.27 |
| **Montenegro** | -0.27 | -0.48 | -0.06 | 0.04 | -0.17 | 0.24 | 0.23 | 0.11 | 0.34 |
| **Morocco** | -0.43 | -0.65 | -0.20 | -0.59 | -0.69 | -0.49 | 1.39 | 1.24 | 1.53 |
| **Mozambique** | 0.91 | 0.76 | 1.06 | 0.88 | 0.74 | 1.01 | -0.74 | -0.82 | -0.66 |
| **Myanmar** | -2.12 | -2.20 | -2.04 | -1.78 | -1.90 | -1.65 | 0.57 | 0.34 | 0.80 |
| **Namibia** | 0.16 | -0.09 | 0.41 | -0.33 | -0.63 | -0.02 | -0.69 | -0.78 | -0.59 |
| **Nauru** | -0.65 | -0.96 | -0.34 | -0.59 | -0.96 | -0.22 | 0.90 | 0.78 | 1.02 |
| **Nepal** | -0.55 | -0.87 | -0.24 | -0.81 | -1.16 | -0.46 | 0.57 | 0.24 | 0.90 |
| **Netherlands** | -2.36 | -2.60 | -2.13 | -1.16 | -1.33 | -1.00 | -0.58 | -0.73 | -0.43 |
| **New Zealand** | -2.44 | -2.56 | -2.32 | -1.40 | -1.62 | -1.19 | -0.43 | -0.70 | -0.17 |
| **Nicaragua** | -1.00 | -1.21 | -0.78 | 0.47 | 0.32 | 0.62 | -0.23 | -0.35 | -0.12 |
| **Niger** | 0.40 | 0.24 | 0.57 | -0.51 | -0.60 | -0.42 | 0.82 | 0.67 | 0.96 |
| **Nigeria** | -0.27 | -0.46 | -0.07 | -0.09 | -0.16 | -0.03 | -0.46 | -0.50 | -0.41 |
| **Niue** | -1.04 | -1.16 | -0.91 | -0.81 | -0.85 | -0.77 | 0.49 | 0.31 | 0.67 |
| **North Macedonia** | -0.31 | -0.58 | -0.05 | -0.70 | -0.83 | -0.58 | 0.74 | 0.28 | 1.20 |
| **Northern Mariana Islands** | -3.12 | -3.67 | -2.57 | 0.91 | 0.64 | 1.18 | -0.61 | -0.78 | -0.44 |
| **Norway** | -2.20 | -2.30 | -2.10 | -1.81 | -1.97 | -1.65 | -0.12 | -0.36 | 0.13 |
| **Oman** | -1.09 | -1.32 | -0.86 | -2.09 | -2.28 | -1.90 | 0.58 | 0.36 | 0.79 |
| **Pakistan** | -0.40 | -0.66 | -0.14 | -0.39 | -0.54 | -0.23 | -0.34 | -0.42 | -0.27 |
| **Palau** | -0.72 | -0.80 | -0.64 | -0.52 | -0.59 | -0.45 | -0.76 | -0.95 | -0.57 |
| **Palestine** | -1.74 | -1.89 | -1.59 | -0.71 | -0.90 | -0.52 | -1.15 | -1.42 | -0.89 |
| **Panama** | -2.58 | -2.77 | -2.39 | 0.37 | 0.13 | 0.62 | 0.34 | 0.30 | 0.38 |
| **Papua New Guinea** | -0.26 | -0.29 | -0.24 | 0.24 | 0.18 | 0.31 | 0.41 | 0.27 | 0.56 |
| **Paraguay** | 0.75 | 0.63 | 0.87 | 0.20 | -0.06 | 0.47 | -2.35 | -2.75 | -1.94 |
| **Peru** | -3.00 | -3.35 | -2.65 | -0.92 | -1.09 | -0.76 | -1.29 | -1.54 | -1.04 |
| **Philippines** | -1.12 | -1.31 | -0.93 | -0.98 | -1.14 | -0.83 | 1.18 | 1.00 | 1.37 |
| **Poland** | -2.00 | -2.12 | -1.87 | -0.23 | -0.57 | 0.12 | 2.40 | 2.03 | 2.78 |
| **Portugal** | -2.29 | -2.48 | -2.09 | -1.30 | -1.47 | -1.12 | -3.33 | -3.68 | -2.98 |
| **Puerto Rico** | -4.19 | -4.51 | -3.88 | -1.93 | -2.11 | -1.74 | 2.01 | 1.62 | 2.41 |
| **Qatar** | -1.61 | -2.03 | -1.19 | -1.06 | -1.26 | -0.86 | 1.87 | 1.32 | 2.43 |
| **Republic of Korea** | -6.51 | -7.11 | -5.90 | -1.87 | -1.97 | -1.77 | 1.80 | 1.41 | 2.18 |
| **Republic of Moldova** | -0.65 | -1.00 | -0.30 | 0.12 | -0.50 | 0.74 | 3.23 | 2.97 | 3.49 |
| **Romania** | -0.22 | -0.43 | -0.01 | 3.03 | 2.23 | 3.83 | -0.53 | -0.83 | -0.24 |
| **Russian Federation** | -2.83 | -3.20 | -2.45 | -1.53 | -1.87 | -1.18 | -1.71 | -2.01 | -1.40 |
| **Rwanda** | -3.18 | -3.56 | -2.79 | -2.48 | -2.81 | -2.16 | -1.91 | -2.36 | -1.45 |
| **Saint Kitts and Nevis** | -0.44 | -0.67 | -0.22 | -0.15 | -0.31 | 0.01 | -1.67 | -2.11 | -1.22 |
| **Saint Lucia** | -0.58 | -0.77 | -0.39 | -0.06 | -0.19 | 0.08 | -0.58 | -1.01 | -0.16 |
| **Saint Vincent and the Grenadines** | 0.56 | 0.36 | 0.76 | 0.52 | 0.38 | 0.66 | -0.46 | -0.60 | -0.33 |
| **Samoa** | -0.91 | -0.96 | -0.87 | -0.75 | -0.87 | -0.64 | -0.01 | -0.10 | 0.07 |
| **San Marino** | -1.60 | -1.86 | -1.34 | -0.92 | -1.06 | -0.79 | 0.93 | 0.85 | 1.00 |
| **Sao Tome and Principe** | 0.96 | 0.85 | 1.07 | 0.40 | 0.26 | 0.54 | -0.42 | -0.80 | -0.03 |
| **Saudi Arabia** | -1.35 | -1.53 | -1.18 | -2.06 | -2.22 | -1.90 | 0.14 | -0.04 | 0.33 |
| **Senegal** | 0.44 | 0.17 | 0.71 | 1.01 | 0.78 | 1.23 | 0.12 | -0.01 | 0.25 |
| **Serbia** | -1.18 | -1.45 | -0.92 | -0.33 | -0.46 | -0.20 | 1.39 | 1.10 | 1.68 |
| **Seychelles** | -0.28 | -0.61 | 0.04 | -0.34 | -0.72 | 0.05 | 0.08 | -0.07 | 0.24 |
| **Sierra Leone** | 0.35 | 0.14 | 0.55 | 1.13 | 0.98 | 1.27 | -1.83 | -2.08 | -1.58 |
| **Singapore** | -5.22 | -5.48 | -4.95 | -4.30 | -4.60 | -4.00 | 0.63 | 0.40 | 0.87 |
| **Slovakia** | -2.63 | -2.73 | -2.53 | -2.76 | -2.89 | -2.63 | -0.41 | -0.61 | -0.21 |
| **Slovenia** | -2.85 | -3.01 | -2.69 | -3.12 | -3.38 | -2.87 | 0.01 | -0.07 | 0.09 |
| **Solomon Islands** | -0.41 | -0.46 | -0.35 | 0.04 | -0.03 | 0.11 | -0.53 | -0.56 | -0.51 |
| **Somalia** | -0.89 | -0.91 | -0.86 | -0.49 | -0.52 | -0.47 | 0.16 | -0.12 | 0.43 |
| **South Africa** | -1.41 | -1.97 | -0.86 | -1.02 | -1.42 | -0.62 | -0.84 | -0.90 | -0.79 |
| **South Sudan** | -1.21 | -1.27 | -1.15 | -0.89 | -0.98 | -0.79 | -0.80 | -1.00 | -0.60 |
| **Spain** | -3.59 | -3.74 | -3.45 | -2.62 | -2.79 | -2.45 | -1.77 | -2.12 | -1.42 |
| **Sri Lanka** | 1.19 | 0.75 | 1.63 | -0.41 | -0.70 | -0.12 | -0.33 | -0.42 | -0.25 |
| **Sudan** | -0.27 | -0.32 | -0.22 | -1.45 | -1.52 | -1.39 | -0.76 | -1.13 | -0.40 |
| **Suriname** | 0.13 | -0.18 | 0.43 | 0.80 | 0.55 | 1.06 | 0.30 | 0.18 | 0.42 |
| **Sweden** | -1.60 | -1.68 | -1.51 | -1.65 | -1.78 | -1.52 | 1.53 | 0.88 | 2.19 |
| **Switzerland** | -3.15 | -3.41 | -2.89 | -0.82 | -1.34 | -0.29 | -0.23 | -0.33 | -0.13 |
| **Syrian Arab Republic** | -0.92 | -1.20 | -0.63 | -0.89 | -1.00 | -0.78 | 2.90 | 2.58 | 3.22 |
| **Taiwan (Province of China)** | -2.34 | -2.61 | -2.07 | -2.83 | -3.13 | -2.54 | 0.14 | -0.06 | 0.33 |
| **Tajikistan** | -1.89 | -2.12 | -1.65 | -0.08 | -0.22 | 0.05 | -1.50 | -1.76 | -1.25 |
| **Thailand** | -2.50 | -2.73 | -2.26 | -1.03 | -1.21 | -0.84 | 0.30 | -0.01 | 0.60 |
| **Timor-Leste** | -0.37 | -0.68 | -0.06 | -0.35 | -0.59 | -0.10 | -0.20 | -0.27 | -0.13 |
| **Togo** | 0.55 | 0.39 | 0.70 | 0.94 | 0.84 | 1.05 | -0.43 | -0.50 | -0.37 |
| **Tokelau** | -1.30 | -1.36 | -1.25 | -0.75 | -0.78 | -0.72 | 0.16 | 0.06 | 0.26 |
| **Tonga** | -0.57 | -0.81 | -0.33 | 0.14 | 0.04 | 0.24 | -2.48 | -2.83 | -2.14 |
| **Trinidad and Tobago** | -1.74 | -1.95 | -1.53 | 0.14 | -0.10 | 0.38 | -0.18 | -0.25 | -0.11 |
| **Tunisia** | -0.88 | -0.98 | -0.79 | -1.20 | -1.29 | -1.10 | -1.62 | -1.81 | -1.42 |
| **Turkey** | -1.93 | -2.20 | -1.66 | -2.53 | -2.65 | -2.41 | -0.33 | -0.61 | -0.05 |
| **Turkmenistan** | -4.13 | -4.59 | -3.67 | 1.35 | 1.06 | 1.65 | -0.57 | -0.66 | -0.48 |
| **Tuvalu** | -0.95 | -1.01 | -0.89 | -0.63 | -0.77 | -0.49 | 0.71 | 0.55 | 0.87 |
| **Uganda** | -0.95 | -1.17 | -0.74 | -1.07 | -1.27 | -0.87 | 1.80 | 1.56 | 2.04 |
| **Ukraine** | -2.79 | -3.27 | -2.31 | 1.38 | 1.02 | 1.74 | -1.16 | -1.50 | -0.82 |
| **United Arab Emirate** | -0.77 | -1.09 | -0.46 | -1.67 | -1.84 | -1.50 | 0.91 | 0.82 | 1.00 |
| **United Kingdom** | -1.78 | -1.90 | -1.66 | -1.27 | -1.38 | -1.16 | -0.13 | -0.21 | -0.04 |
| **United Republic of Tanzania** | -0.92 | -0.96 | -0.88 | -0.62 | -0.70 | -0.54 | -0.34 | -0.54 | -0.15 |
| **United States of America** | -1.80 | -1.92 | -1.68 | -1.63 | -1.78 | -1.49 | 0.21 | -0.13 | 0.55 |
| **United States Virgin Islands** | 1.36 | 0.98 | 1.74 | 0.54 | 0.36 | 0.72 | -1.69 | -1.86 | -1.53 |
| **Uruguay** | -2.13 | -2.28 | -1.98 | -1.38 | -1.63 | -1.12 | 2.57 | 2.34 | 2.80 |
| **Uzbekistan** | -1.36 | -2.00 | -0.71 | 1.74 | 1.50 | 1.97 | -0.05 | -0.19 | 0.10 |
| **Vanuatu** | -0.59 | -0.73 | -0.45 | -0.01 | -0.15 | 0.13 | -0.48 | -0.91 | -0.05 |
| **Venezuela (Bolivarian Republic of)** | -0.89 | -1.14 | -0.64 | 0.60 | 0.34 | 0.86 | 1.08 | 0.97 | 1.18 |
| **Viet Nam** | 0.94 | 0.87 | 1.00 | -0.63 | -0.71 | -0.55 | -0.19 | -0.24 | -0.14 |
| **Yemen** | 0.08 | 0.03 | 0.14 | -1.14 | -1.23 | -1.04 | -0.32 | -0.42 | -0.22 |
| **Zambia** | -1.01 | -1.26 | -0.76 | -0.92 | -1.07 | -0.76 | 1.09 | 0.76 | 1.42 |
| **Zimbabwe** | -0.03 | -0.15 | 0.08 | 1.07 | 0.82 | 1.32 | -1.45 | -1.81 | -1.09 |

**Supporting Table 2.** The global mortality, age-standardized mortality rate, and disability-adjusted life-years (DALYs) due to pharynx and larynx cancers in 1990 and 2019.

|  | | **Larynx cancer** | **Other pharynx cancer** | **Nasopharynx cancer** |
| --- | --- | --- | --- | --- |
| DALYs ×10^6^ | | 2.47 (2.35 to 2.59) * | 1.56 (1.45 to 1.71) | 1.88 (1.72 to 2.05) |
|  |  | 3.26 (3.03 to 3.51) | 3.23 (2.90 to 3.57) | 2.34 (2.14 to 2.54) |
| Deaths Number  ×10^3^ | | 87.46 (83.18 to 91.55) | 51.46 (47.97 to 56.46) | 53.46 (48.87 to 57.91) |
|  |  | 123.34 (114.94 to 132.80) | 114.21 (103.15 to 126.04) | 71.61 (65.44 to 77.62) |
| ASMR  per100,000 | Both sex | 2.19 (2.08 to 2.29) | 1.25 (1.17 to 1.37) | 1.26 (1.15 to 1.36) |
|  |  | 1.49 (1.39 to 1.61) | 1.37 (1.24 to 1.51) | 0.86 (0.79 to 0.93) |
|  | male | 4.15 (3.94 to 4.35) | 2.04 (1.86 to 2.25) | 1.72 (1.55 to 1.90) |
|  |  | 2.74 (2.54 to 2.98) | 2.23 (1.98 to 2.50) | 1.28 (1.15 to 1.43) |
|  | female | 0.54 (0.50 to 0.58) | 0.57 (0.49 to 0.65) | 0.83 (0.71 to 0.94) |
|  |  | 0.41 (0.37 to 0.45) | 0.60 (0.52 to 0.70) | 0.47 (0.42 to 0.53) |

ASMR, age-standardized mortality rate.

* Data in parentheses shown as 95% uncertainty interval, data for 1990 are presented in the above column

**Supporting Table 3.** The age-standardized mortality rate of larynx cancer, nasopharynx cancer, and other pharynx cancer for different countries and regions in 2019.

| **location** | **Larynx cancer** | | | **Other pharynx**  **cancer** | | | **Nasopharynx cancer** | | |
| --- | --- | --- | --- | --- | --- | --- | --- | --- | --- |
|  | **Estimate value** | **Lower 95%UI** | **Upper 95%UI** | **Estimate value** | **Lower 95%UI** | **Upper 95%UI** | **Estimate value** | **Lower 95%UI** | **Upper 95%UI** |
| **Afghanistan** | 3.26 | 4.22 | 2.32 | 0.42 | 0.64 | 0.28 | 0.59 | 0.82 | 0.39 |
| **Albania** | 2.05 | 2.76 | 1.49 | 0.60 | 0.80 | 0.43 | 0.36 | 0.46 | 0.27 |
| **Algeria** | 1.39 | 1.76 | 1.08 | 0.61 | 0.77 | 0.47 | 1.80 | 2.26 | 1.41 |
| **American Samoa** | 0.58 | 0.68 | 0.49 | 0.42 | 0.51 | 0.35 | 1.59 | 1.94 | 1.32 |
| **Andorra** | 1.01 | 1.31 | 0.76 | 0.88 | 1.14 | 0.64 | 0.29 | 0.38 | 0.21 |
| **Angola** | 1.62 | 2.06 | 1.29 | 0.60 | 0.77 | 0.45 | 0.54 | 0.71 | 0.40 |
| **Antigua and Barbuda** | 1.60 | 1.92 | 1.34 | 1.06 | 1.27 | 0.87 | 0.39 | 0.47 | 0.32 |
| **Argentina** | 1.97 | 2.15 | 1.80 | 0.44 | 0.49 | 0.40 | 0.21 | 0.23 | 0.18 |
| **Armenia** | 2.53 | 3.04 | 2.09 | 0.37 | 0.45 | 0.30 | 0.21 | 0.26 | 0.18 |
| **Australia** | 0.61 | 0.67 | 0.55 | 0.88 | 0.99 | 0.77 | 0.30 | 0.34 | 0.27 |
| **Austria** | 0.86 | 0.95 | 0.79 | 1.41 | 1.57 | 1.26 | 0.18 | 0.21 | 0.16 |
| **Azerbaijan** | 2.42 | 3.23 | 1.93 | 0.38 | 0.50 | 0.30 | 0.18 | 0.22 | 0.14 |
| **Bahamas** | 2.47 | 3.05 | 1.98 | 1.58 | 1.98 | 1.25 | 0.53 | 0.65 | 0.44 |
| **Bahrain** | 1.18 | 1.52 | 0.84 | 0.30 | 0.39 | 0.23 | 0.33 | 0.42 | 0.25 |
| **Bangladesh** | 2.25 | 3.21 | 1.67 | 3.35 | 4.85 | 2.31 | 0.87 | 1.22 | 0.62 |
| **Barbados** | 1.42 | 1.71 | 1.17 | 1.37 | 1.67 | 1.09 | 0.62 | 0.75 | 0.51 |
| **Belarus** | 2.19 | 2.88 | 1.67 | 1.89 | 2.54 | 1.40 | 0.33 | 0.43 | 0.24 |
| **Belgium** | 1.12 | 1.23 | 1.02 | 1.13 | 1.25 | 1.02 | 0.28 | 0.31 | 0.25 |
| **Belize** | 1.70 | 1.98 | 1.47 | 0.64 | 0.76 | 0.54 | 0.36 | 0.42 | 0.31 |
| **Benin** | 1.24 | 1.59 | 0.95 | 0.31 | 0.41 | 0.24 | 0.28 | 0.39 | 0.21 |
| **Bermuda** | 1.59 | 1.92 | 1.33 | 1.07 | 1.31 | 0.86 | 0.45 | 0.56 | 0.36 |
| **Bhutan** | 2.15 | 3.07 | 1.50 | 4.02 | 5.51 | 2.51 | 0.86 | 1.18 | 0.60 |
| **Bolivia (Plurinational State of)** | 1.32 | 1.67 | 1.01 | 0.42 | 0.54 | 0.32 | 0.26 | 0.33 | 0.19 |
| **Bosnia and Herzegovina** | 2.56 | 3.27 | 2.00 | 1.12 | 1.43 | 0.86 | 0.20 | 0.25 | 0.16 |
| **Botswana** | 2.13 | 2.83 | 1.55 | 0.98 | 1.33 | 0.67 | 0.41 | 0.55 | 0.29 |
| **Brazil** | 2.27 | 2.39 | 2.14 | 1.60 | 1.70 | 1.48 | 0.22 | 0.23 | 0.20 |
| **Brunei Darussalam** | 1.08 | 1.29 | 0.92 | 2.08 | 2.48 | 1.73 | 2.91 | 3.41 | 2.48 |
| **Bulgaria** | 3.06 | 3.91 | 2.34 | 1.29 | 1.67 | 0.99 | 0.37 | 0.47 | 0.28 |
| **Burkina Faso** | 1.48 | 1.85 | 1.16 | 0.32 | 0.42 | 0.25 | 0.32 | 0.42 | 0.24 |
| **Burundi** | 1.52 | 2.08 | 1.10 | 0.81 | 1.21 | 0.53 | 1.40 | 1.92 | 0.95 |
| **Cabo Verde** | 1.37 | 1.63 | 1.17 | 0.59 | 0.74 | 0.48 | 0.30 | 0.37 | 0.24 |
| **Cambodia** | 1.64 | 2.12 | 1.24 | 0.63 | 0.79 | 0.49 | 1.98 | 2.50 | 1.48 |
| **Cameroon** | 1.62 | 2.13 | 1.21 | 0.40 | 0.55 | 0.30 | 0.95 | 1.34 | 0.67 |
| **Canada** | 0.73 | 0.80 | 0.66 | 0.60 | 0.67 | 0.52 | 0.22 | 0.25 | 0.20 |
| **Central African Republic** | 1.79 | 2.40 | 1.33 | 0.53 | 0.77 | 0.37 | 0.68 | 0.92 | 0.49 |
| **Chad** | 1.39 | 1.75 | 1.08 | 0.35 | 0.46 | 0.26 | 0.32 | 0.41 | 0.24 |
| **Chile** | 0.74 | 0.82 | 0.67 | 0.38 | 0.42 | 0.33 | 0.11 | 0.13 | 0.10 |
| **China** | 1.02 | 1.21 | 0.85 | 0.28 | 0.33 | 0.23 | 1.43 | 1.69 | 1.19 |
| **Colombia** | 1.03 | 1.32 | 0.79 | 0.26 | 0.34 | 0.20 | 0.17 | 0.23 | 0.13 |
| **Comoros** | 1.02 | 1.34 | 0.79 | 0.72 | 1.05 | 0.48 | 1.12 | 1.58 | 0.80 |
| **Congo** | 1.55 | 1.97 | 1.22 | 0.65 | 0.95 | 0.48 | 0.57 | 0.78 | 0.41 |
| **Cook Islands** | 0.51 | 0.62 | 0.42 | 0.21 | 0.26 | 0.17 | 0.21 | 0.25 | 0.17 |
| **Costa Rica** | 1.10 | 1.40 | 0.84 | 0.53 | 0.70 | 0.40 | 0.38 | 0.50 | 0.29 |
| **Croatia** | 2.29 | 2.95 | 1.77 | 1.94 | 2.56 | 1.44 | 0.23 | 0.31 | 0.17 |
| **Cuba** | 5.03 | 6.15 | 4.08 | 1.18 | 1.48 | 0.93 | 0.52 | 0.66 | 0.41 |
| **Cyprus** | 0.81 | 0.95 | 0.68 | 0.25 | 0.30 | 0.21 | 0.16 | 0.19 | 0.13 |
| **Czechia** | 1.35 | 1.68 | 1.09 | 1.58 | 2.01 | 1.24 | 0.21 | 0.27 | 0.17 |
| **Côte d'Ivoire** | 1.56 | 1.96 | 1.18 | 0.38 | 0.49 | 0.29 | 0.32 | 0.43 | 0.23 |
| **Democratic People's Republic of Korea** | 0.99 | 1.22 | 0.79 | 0.27 | 0.37 | 0.21 | 1.60 | 2.05 | 1.21 |
| **Democratic Republic of the Congo** | 1.18 | 1.54 | 0.90 | 0.42 | 0.62 | 0.26 | 0.49 | 0.65 | 0.36 |
| **Denmark** | 0.92 | 1.01 | 0.83 | 1.47 | 1.70 | 1.26 | 0.14 | 0.16 | 0.12 |
| **Djibouti** | 1.38 | 1.94 | 1.01 | 0.96 | 1.43 | 0.66 | 1.28 | 1.82 | 0.86 |
| **Dominica** | 2.41 | 2.98 | 1.89 | 2.01 | 2.55 | 1.56 | 0.60 | 0.76 | 0.49 |
| **Dominican Republic** | 2.08 | 2.67 | 1.60 | 1.66 | 2.22 | 1.22 | 0.46 | 0.59 | 0.35 |
| **Ecuador** | 0.75 | 0.96 | 0.59 | 0.25 | 0.33 | 0.20 | 0.18 | 0.23 | 0.14 |
| **Egypt** | 1.00 | 1.43 | 0.75 | 0.24 | 0.32 | 0.17 | 0.11 | 0.15 | 0.08 |
| **El Salvador** | 0.78 | 1.01 | 0.59 | 0.33 | 0.43 | 0.25 | 0.20 | 0.26 | 0.15 |
| **Equatorial Guinea** | 1.17 | 1.76 | 0.82 | 0.66 | 0.97 | 0.40 | 0.44 | 0.70 | 0.26 |
| **Eritrea** | 1.38 | 1.85 | 1.00 | 0.88 | 1.17 | 0.63 | 1.41 | 1.93 | 0.95 |
| **Estonia** | 1.33 | 1.70 | 1.01 | 1.67 | 2.19 | 1.24 | 0.27 | 0.35 | 0.19 |
| **Eswatini** | 2.17 | 2.91 | 1.64 | 0.88 | 1.19 | 0.62 | 0.72 | 0.99 | 0.51 |
| **Ethiopia** | 0.93 | 1.27 | 0.74 | 0.34 | 0.50 | 0.23 | 1.03 | 1.35 | 0.64 |
| **Fiji** | 0.80 | 1.00 | 0.62 | 0.40 | 0.50 | 0.30 | 0.37 | 0.48 | 0.29 |
| **Finland** | 0.38 | 0.43 | 0.34 | 0.54 | 0.62 | 0.47 | 0.10 | 0.11 | 0.09 |
| **France** | 1.46 | 1.61 | 1.32 | 1.78 | 2.03 | 1.54 | 0.34 | 0.39 | 0.30 |
| **Gabon** | 1.74 | 2.36 | 1.30 | 0.79 | 1.07 | 0.55 | 0.56 | 0.75 | 0.39 |
| **Gambia** | 0.69 | 0.85 | 0.54 | 0.31 | 0.42 | 0.22 | 0.23 | 0.30 | 0.17 |
| **Georgia** | 3.48 | 4.23 | 2.84 | 0.98 | 1.18 | 0.81 | 0.29 | 0.35 | 0.24 |
| **Germany** | 0.93 | 1.02 | 0.85 | 1.74 | 1.94 | 1.54 | 0.17 | 0.19 | 0.15 |
| **Ghana** | 1.54 | 1.91 | 1.21 | 0.25 | 0.31 | 0.20 | 0.15 | 0.20 | 0.12 |
| **Greece** | 1.73 | 1.87 | 1.59 | 0.25 | 0.27 | 0.23 | 0.41 | 0.44 | 0.37 |
| **Greenland** | 1.46 | 1.77 | 1.15 | 3.00 | 3.73 | 2.30 | 4.68 | 5.73 | 3.76 |
| **Grenada** | 1.25 | 1.41 | 1.10 | 1.45 | 1.69 | 1.25 | 0.57 | 0.64 | 0.50 |
| **Guam** | 0.38 | 0.49 | 0.31 | 0.40 | 0.50 | 0.32 | 2.51 | 3.09 | 2.01 |
| **Guatemala** | 0.75 | 0.94 | 0.61 | 0.37 | 0.46 | 0.29 | 0.25 | 0.31 | 0.20 |
| **Guinea** | 1.38 | 1.81 | 1.01 | 0.48 | 0.64 | 0.36 | 0.30 | 0.38 | 0.23 |
| **Guinea-Bissau** | 1.83 | 2.33 | 1.30 | 0.40 | 0.54 | 0.29 | 0.43 | 0.60 | 0.30 |
| **Guyana** | 1.23 | 1.57 | 0.94 | 0.67 | 0.88 | 0.50 | 0.33 | 0.43 | 0.25 |
| **Haiti** | 2.47 | 3.44 | 1.49 | 1.05 | 1.70 | 0.67 | 0.77 | 1.05 | 0.50 |
| **Honduras** | 1.35 | 1.64 | 1.05 | 0.58 | 0.78 | 0.44 | 0.16 | 0.20 | 0.12 |
| **Hungary** | 2.80 | 3.44 | 2.27 | 3.52 | 4.43 | 2.78 | 0.55 | 0.68 | 0.43 |
| **Iceland** | 0.46 | 0.53 | 0.40 | 0.37 | 0.44 | 0.32 | 0.15 | 0.18 | 0.13 |
| **India** | 2.59 | 3.08 | 2.17 | 4.89 | 5.84 | 4.05 | 0.92 | 1.09 | 0.79 |
| **Indonesia** | 1.06 | 1.40 | 0.82 | 0.56 | 0.94 | 0.40 | 1.42 | 1.81 | 1.13 |
| **Iran (Islamic Republic of)** | 2.08 | 2.30 | 1.89 | 0.21 | 0.23 | 0.19 | 0.17 | 0.19 | 0.16 |
| **Iraq** | 2.40 | 2.92 | 1.86 | 0.43 | 0.53 | 0.33 | 0.22 | 0.28 | 0.17 |
| **Ireland** | 0.83 | 0.93 | 0.74 | 0.73 | 0.84 | 0.63 | 0.18 | 0.21 | 0.15 |
| **Israel** | 0.83 | 0.91 | 0.75 | 0.19 | 0.22 | 0.17 | 0.18 | 0.20 | 0.16 |
| **Italy** | 1.21 | 1.29 | 1.13 | 0.72 | 0.78 | 0.67 | 0.28 | 0.29 | 0.26 |
| **Jamaica** | 1.30 | 1.67 | 1.00 | 0.63 | 0.80 | 0.47 | 0.51 | 0.65 | 0.40 |
| **Japan** | 0.31 | 0.33 | 0.28 | 0.81 | 0.87 | 0.75 | 0.28 | 0.30 | 0.26 |
| **Jordan** | 0.74 | 0.98 | 0.57 | 0.30 | 0.37 | 0.24 | 0.44 | 0.55 | 0.36 |
| **Kazakhstan** | 1.49 | 1.74 | 1.26 | 0.91 | 1.10 | 0.77 | 0.44 | 0.52 | 0.37 |
| **Kenya** | 1.41 | 1.92 | 1.10 | 0.92 | 1.24 | 0.68 | 1.71 | 2.25 | 1.12 |
| **Kiribati** | 0.75 | 0.93 | 0.60 | 0.62 | 0.79 | 0.47 | 1.36 | 1.82 | 1.00 |
| **Kuwait** | 0.72 | 0.87 | 0.57 | 0.26 | 0.33 | 0.20 | 0.24 | 0.31 | 0.19 |
| **Kyrgyzstan** | 0.83 | 0.98 | 0.70 | 0.66 | 0.78 | 0.54 | 0.38 | 0.45 | 0.32 |
| **Lao People's Democratic Republic** | 1.23 | 1.64 | 0.90 | 0.55 | 0.71 | 0.40 | 1.61 | 2.08 | 1.15 |
| **Latvia** | 1.92 | 2.45 | 1.48 | 1.71 | 2.27 | 1.27 | 0.26 | 0.34 | 0.20 |
| **Lebanon** | 2.89 | 3.71 | 2.36 | 0.37 | 0.48 | 0.29 | 0.44 | 0.58 | 0.34 |
| **Lesotho** | 2.75 | 3.57 | 2.12 | 0.95 | 1.24 | 0.70 | 0.95 | 1.26 | 0.67 |
| **Liberia** | 1.26 | 1.67 | 0.94 | 0.29 | 0.41 | 0.18 | 0.29 | 0.40 | 0.21 |
| **Libya** | 2.77 | 3.56 | 2.13 | 0.26 | 0.34 | 0.19 | 2.23 | 2.86 | 1.73 |
| **Lithuania** | 2.23 | 2.79 | 1.78 | 2.33 | 2.99 | 1.82 | 0.26 | 0.33 | 0.19 |
| **Luxembourg** | 0.95 | 1.11 | 0.83 | 1.11 | 1.34 | 0.93 | 0.32 | 0.38 | 0.27 |
| **Madagascar** | 0.99 | 1.30 | 0.74 | 0.65 | 0.94 | 0.45 | 1.03 | 1.43 | 0.71 |
| **Malawi** | 0.53 | 0.68 | 0.43 | 0.21 | 0.28 | 0.15 | 0.28 | 0.38 | 0.21 |
| **Malaysia** | 1.30 | 1.65 | 1.00 | 0.85 | 1.09 | 0.65 | 4.76 | 6.08 | 3.65 |
| **Maldives** | 0.83 | 1.03 | 0.67 | 0.74 | 0.92 | 0.58 | 0.20 | 0.24 | 0.16 |
| **Mali** | 1.00 | 1.28 | 0.77 | 0.31 | 0.42 | 0.23 | 0.14 | 0.19 | 0.11 |
| **Malta** | 0.99 | 1.14 | 0.86 | 0.51 | 0.60 | 0.45 | 0.72 | 0.83 | 0.61 |
| **Marshall Islands** | 1.21 | 1.59 | 0.87 | 0.43 | 0.62 | 0.31 | 1.76 | 2.54 | 1.22 |
| **Mauritania** | 0.95 | 1.24 | 0.70 | 0.30 | 0.48 | 0.20 | 0.22 | 0.33 | 0.15 |
| **Mauritius** | 1.11 | 1.37 | 0.88 | 0.56 | 0.70 | 0.44 | 0.66 | 0.83 | 0.53 |
| **Mexico** | 0.99 | 1.18 | 0.82 | 0.24 | 0.28 | 0.21 | 0.15 | 0.17 | 0.12 |
| **Micronesia (Federated States of)** | 1.10 | 1.42 | 0.81 | 0.41 | 0.61 | 0.29 | 1.60 | 2.25 | 1.08 |
| **Monaco** | 3.24 | 3.96 | 2.57 | 0.43 | 0.54 | 0.32 | 0.21 | 0.27 | 0.17 |
| **Mongolia** | 1.72 | 2.19 | 1.24 | 0.65 | 0.82 | 0.51 | 0.29 | 0.37 | 0.21 |
| **Montenegro** | 4.74 | 5.81 | 3.86 | 0.71 | 0.88 | 0.56 | 0.12 | 0.15 | 0.10 |
| **Morocco** | 3.01 | 3.73 | 2.21 | 0.64 | 0.81 | 0.48 | 1.99 | 2.53 | 1.48 |
| **Mozambique** | 1.71 | 2.18 | 1.33 | 0.61 | 0.80 | 0.46 | 0.14 | 0.18 | 0.11 |
| **Myanmar** | 1.07 | 1.39 | 0.87 | 0.49 | 0.61 | 0.39 | 1.43 | 1.77 | 1.17 |
| **Namibia** | 2.76 | 3.52 | 2.16 | 1.46 | 1.92 | 1.10 | 0.53 | 0.70 | 0.40 |
| **Nauru** | 1.33 | 1.68 | 1.03 | 0.46 | 0.60 | 0.33 | 1.60 | 2.21 | 1.10 |
| **Nepal** | 2.51 | 3.33 | 1.91 | 3.55 | 4.82 | 2.56 | 0.98 | 1.23 | 0.73 |
| **Netherlands** | 0.79 | 0.86 | 0.71 | 0.78 | 0.88 | 0.68 | 0.24 | 0.27 | 0.21 |
| **New Zealand** | 0.47 | 0.51 | 0.42 | 0.55 | 0.61 | 0.49 | 0.29 | 0.32 | 0.27 |
| **Nicaragua** | 1.27 | 1.58 | 1.01 | 0.41 | 0.50 | 0.32 | 0.28 | 0.34 | 0.23 |
| **Niger** | 1.09 | 1.39 | 0.80 | 0.25 | 0.36 | 0.18 | 0.11 | 0.15 | 0.08 |
| **Nigeria** | 1.17 | 1.60 | 0.86 | 0.21 | 0.28 | 0.17 | 0.84 | 1.07 | 0.63 |
| **Niue** | 0.77 | 0.94 | 0.63 | 0.34 | 0.43 | 0.27 | 1.02 | 1.32 | 0.77 |
| **North Macedonia** | 3.65 | 4.62 | 2.83 | 0.77 | 0.99 | 0.59 | 0.27 | 0.34 | 0.21 |
| **Northern Mariana Islands** | 0.65 | 0.81 | 0.55 | 1.26 | 1.50 | 1.03 | 2.56 | 3.00 | 2.12 |
| **Norway** | 0.41 | 0.45 | 0.39 | 0.51 | 0.56 | 0.46 | 0.10 | 0.11 | 0.10 |
| **Oman** | 0.69 | 0.83 | 0.58 | 0.37 | 0.49 | 0.29 | 0.29 | 0.36 | 0.23 |
| **Pakistan** | 5.75 | 7.44 | 4.47 | 3.79 | 4.91 | 2.95 | 1.35 | 1.70 | 1.08 |
| **Palau** | 0.56 | 0.71 | 0.44 | 0.20 | 0.26 | 0.16 | 0.23 | 0.30 | 0.18 |
| **Palestine** | 0.98 | 1.17 | 0.82 | 0.19 | 0.22 | 0.16 | 0.26 | 0.31 | 0.21 |
| **Panama** | 1.03 | 1.34 | 0.77 | 0.59 | 0.77 | 0.43 | 0.29 | 0.38 | 0.22 |
| **Papua New Guinea** | 0.85 | 1.12 | 0.64 | 0.30 | 0.45 | 0.22 | 1.26 | 1.71 | 0.89 |
| **Paraguay** | 1.52 | 1.98 | 1.13 | 1.01 | 1.33 | 0.73 | 0.16 | 0.21 | 0.12 |
| **Peru** | 0.57 | 0.76 | 0.42 | 0.22 | 0.29 | 0.16 | 0.12 | 0.16 | 0.09 |
| **Philippines** | 0.91 | 1.13 | 0.73 | 0.45 | 0.54 | 0.37 | 1.69 | 2.04 | 1.38 |
| **Poland** | 2.63 | 3.26 | 2.10 | 1.58 | 1.95 | 1.28 | 0.35 | 0.42 | 0.29 |
| **Portugal** | 1.84 | 2.01 | 1.66 | 1.64 | 1.84 | 1.43 | 0.41 | 0.46 | 0.36 |
| **Puerto Rico** | 0.91 | 1.17 | 0.69 | 0.67 | 0.88 | 0.50 | 0.33 | 0.43 | 0.25 |
| **Qatar** | 2.93 | 3.97 | 2.00 | 0.66 | 0.95 | 0.45 | 0.34 | 0.47 | 0.24 |
| **Republic of Korea** | 0.58 | 0.68 | 0.51 | 0.55 | 0.64 | 0.46 | 0.23 | 0.26 | 0.20 |
| **Republic of Moldova** | 2.41 | 2.80 | 2.05 | 2.50 | 3.01 | 2.09 | 0.57 | 0.69 | 0.48 |
| **Romania** | 3.05 | 3.70 | 2.43 | 2.84 | 3.49 | 2.21 | 0.70 | 0.85 | 0.55 |
| **Russian Federation** | 1.76 | 2.10 | 1.45 | 1.26 | 1.53 | 1.04 | 0.25 | 0.29 | 0.21 |
| **Rwanda** | 1.32 | 1.76 | 1.03 | 0.80 | 1.09 | 0.57 | 1.19 | 1.61 | 0.83 |
| **Saint Kitts and Nevis** | 1.68 | 1.97 | 1.43 | 1.06 | 1.27 | 0.88 | 0.83 | 1.00 | 0.68 |
| **Saint Lucia** | 2.13 | 2.51 | 1.79 | 1.09 | 1.32 | 0.89 | 0.72 | 0.86 | 0.61 |
| **Saint Vincent and the Grenadines** | 2.87 | 3.33 | 2.51 | 1.51 | 1.77 | 1.30 | 0.65 | 0.76 | 0.57 |
| **Samoa** | 0.77 | 0.93 | 0.64 | 0.36 | 0.44 | 0.28 | 1.58 | 2.10 | 1.21 |
| **San Marino** | 1.33 | 1.94 | 0.85 | 0.54 | 0.82 | 0.34 | 0.55 | 0.82 | 0.35 |
| **Sao Tome and Principe** | 1.19 | 1.45 | 0.96 | 0.29 | 0.38 | 0.22 | 0.15 | 0.18 | 0.12 |
| **Saudi Arabia** | 0.64 | 0.84 | 0.51 | 0.32 | 0.42 | 0.25 | 0.80 | 1.00 | 0.62 |
| **Senegal** | 1.22 | 1.57 | 0.95 | 0.34 | 0.44 | 0.25 | 0.29 | 0.40 | 0.21 |
| **Serbia** | 2.93 | 3.72 | 2.25 | 1.68 | 2.17 | 1.26 | 0.35 | 0.45 | 0.27 |
| **Seychelles** | 5.12 | 6.06 | 4.34 | 3.92 | 4.84 | 3.16 | 1.74 | 2.07 | 1.46 |
| **Sierra Leone** | 1.33 | 1.76 | 1.01 | 0.31 | 0.42 | 0.22 | 0.30 | 0.40 | 0.22 |
| **Singapore** | 0.46 | 0.53 | 0.40 | 0.52 | 0.61 | 0.44 | 1.88 | 2.16 | 1.64 |
| **Slovakia** | 1.95 | 2.54 | 1.48 | 3.47 | 4.56 | 2.59 | 0.34 | 0.45 | 0.26 |
| **Slovenia** | 1.31 | 1.73 | 1.00 | 2.48 | 3.34 | 1.85 | 0.19 | 0.25 | 0.14 |
| **Solomon Islands** | 1.41 | 1.79 | 0.98 | 0.44 | 0.73 | 0.32 | 2.01 | 3.03 | 1.18 |
| **Somalia** | 1.36 | 1.86 | 0.96 | 0.60 | 1.00 | 0.36 | 1.38 | 1.94 | 0.96 |
| **South Africa** | 1.37 | 1.51 | 1.24 | 0.61 | 0.69 | 0.53 | 0.44 | 0.49 | 0.40 |
| **South Sudan** | 0.96 | 1.38 | 0.67 | 0.82 | 1.20 | 0.46 | 0.96 | 1.49 | 0.58 |
| **Spain** | 1.65 | 1.82 | 1.50 | 1.03 | 1.16 | 0.91 | 0.33 | 0.37 | 0.29 |
| **Sri Lanka** | 0.71 | 0.95 | 0.52 | 1.48 | 2.03 | 1.06 | 0.81 | 1.09 | 0.60 |
| **Sudan** | 1.91 | 2.51 | 1.38 | 0.27 | 0.38 | 0.19 | 0.33 | 0.44 | 0.23 |
| **Suriname** | 0.87 | 1.07 | 0.70 | 0.57 | 0.70 | 0.46 | 0.88 | 1.08 | 0.71 |
| **Sweden** | 0.34 | 0.37 | 0.31 | 0.61 | 0.67 | 0.55 | 0.10 | 0.11 | 0.09 |
| **Switzerland** | 0.60 | 0.67 | 0.54 | 1.00 | 1.14 | 0.88 | 0.24 | 0.27 | 0.20 |
| **Syrian Arab Republic** | 0.88 | 1.14 | 0.67 | 0.21 | 0.28 | 0.16 | 0.11 | 0.15 | 0.09 |
| **Taiwan (Province of China)** | 0.70 | 0.91 | 0.54 | 2.41 | 3.21 | 1.82 | 2.43 | 3.20 | 1.85 |
| **Tajikistan** | 0.98 | 1.31 | 0.77 | 0.65 | 0.80 | 0.51 | 0.58 | 0.71 | 0.46 |
| **Thailand** | 1.17 | 1.57 | 0.86 | 0.50 | 0.68 | 0.36 | 1.16 | 1.55 | 0.84 |
| **Timor-Leste** | 1.06 | 1.41 | 0.75 | 0.53 | 0.78 | 0.36 | 1.52 | 2.06 | 1.05 |
| **Togo** | 1.35 | 1.76 | 1.01 | 0.32 | 0.45 | 0.23 | 0.32 | 0.42 | 0.23 |
| **Tokelau** | 0.64 | 0.80 | 0.52 | 0.30 | 0.39 | 0.22 | 0.93 | 1.24 | 0.69 |
| **Tonga** | 0.65 | 0.83 | 0.52 | 0.27 | 0.38 | 0.19 | 0.93 | 1.25 | 0.70 |
| **Trinidad and Tobago** | 0.96 | 1.25 | 0.72 | 0.64 | 0.84 | 0.48 | 0.33 | 0.44 | 0.25 |
| **Tunisia** | 2.42 | 3.33 | 1.73 | 0.48 | 0.66 | 0.35 | 1.74 | 2.38 | 1.25 |
| **Turkey** | 1.65 | 2.06 | 1.30 | 0.21 | 0.26 | 0.16 | 0.46 | 0.57 | 0.36 |
| **Turkmenistan** | 0.97 | 1.21 | 0.77 | 1.03 | 1.33 | 0.80 | 0.37 | 0.47 | 0.30 |
| **Tuvalu** | 0.97 | 1.30 | 0.71 | 0.36 | 0.53 | 0.25 | 1.44 | 2.20 | 0.99 |
| **Uganda** | 1.44 | 1.77 | 1.14 | 1.58 | 2.03 | 1.20 | 2.05 | 2.61 | 1.55 |
| **Ukraine** | 2.40 | 2.97 | 1.88 | 2.26 | 2.85 | 1.74 | 0.49 | 0.60 | 0.40 |
| **United Arab Emirate** | 3.05 | 4.19 | 2.16 | 0.54 | 0.88 | 0.27 | 0.41 | 0.60 | 0.29 |
| **United Kingdom** | 0.77 | 0.79 | 0.72 | 0.85 | 0.89 | 0.81 | 0.25 | 0.26 | 0.24 |
| **United Republic of Tanzania** | 1.29 | 1.80 | 1.00 | 0.88 | 1.22 | 0.62 | 1.23 | 1.72 | 0.83 |
| **United States of America** | 0.88 | 0.92 | 0.84 | 0.57 | 0.60 | 0.55 | 0.22 | 0.23 | 0.21 |
| **United States Virgin Islands** | 2.06 | 2.41 | 1.69 | 1.51 | 1.82 | 1.23 | 0.76 | 0.89 | 0.63 |
| **Uruguay** | 2.65 | 2.93 | 2.39 | 0.96 | 1.12 | 0.83 | 0.34 | 0.38 | 0.30 |
| **Uzbekistan** | 1.72 | 2.01 | 1.45 | 1.19 | 1.40 | 0.99 | 0.55 | 0.66 | 0.45 |
| **Vanuatu** | 1.06 | 1.41 | 0.73 | 0.35 | 0.51 | 0.25 | 1.50 | 2.07 | 1.05 |
| **Venezuela (Bolivarian Republic of)** | 2.20 | 2.88 | 1.67 | 0.54 | 0.71 | 0.40 | 0.30 | 0.39 | 0.22 |
| **Viet Nam** | 1.92 | 2.38 | 1.49 | 2.41 | 3.16 | 1.77 | 2.73 | 3.46 | 2.13 |
| **Yemen** | 2.49 | 3.36 | 1.76 | 0.29 | 0.44 | 0.20 | 0.39 | 0.53 | 0.29 |
| **Zambia** | 1.60 | 2.11 | 1.20 | 1.04 | 1.37 | 0.78 | 1.36 | 1.83 | 0.99 |
| **Zimbabwe** | 2.25 | 2.74 | 1.84 | 0.59 | 0.75 | 0.45 | 0.70 | 0.89 | 0.53 |

UI, uncertainty interval.

**Supporting Table 4.** The SDI values by 204 locations in 2019.

| Location Name | SDI Index Value |
| --- | --- |
| Afghanistan | 0.343 |
| Albania | 0.681 |
| Algeria | 0.652 |
| American Samoa | 0.712 |
| Andorra | 0.894 |
| Angola | 0.47 |
| Antigua and Barbuda | 0.743 |
| Argentina | 0.708 |
| Armenia | 0.689 |
| Australia | 0.839 |
| Austria | 0.849 |
| Azerbaijan | 0.683 |
| Bahamas | 0.796 |
| Bahrain | 0.751 |
| Bangladesh | 0.483 |
| Barbados | 0.742 |
| Belarus | 0.745 |
| Belgium | 0.851 |
| Belize | 0.603 |
| Benin | 0.352 |
| Bermuda | 0.813 |
| Bhutan | 0.455 |
| Bolivia | 0.566 |
| Bosnia and Herzegovina | 0.718 |
| Botswana | 0.634 |
| Brazil | 0.64 |
| Brunei | 0.823 |
| Bulgaria | 0.764 |
| Burkina Faso | 0.257 |
| Burundi | 0.284 |
| Cabo Verde | 0.525 |
| Cambodia | 0.469 |
| Cameroon | 0.49 |
| Canada | 0.873 |
| Central African Republic | 0.274 |
| Chad | 0.238 |
| Chile | 0.759 |
| China | 0.686 |
| Colombia | 0.633 |
| Comoros | 0.455 |
| Congo (Brazzaville) | 0.568 |
| Cook Islands | 0.764 |
| Costa Rica | 0.68 |
| Côte d'Ivoire | 0.408 |
| Croatia | 0.794 |
| Cuba | 0.668 |
| Cyprus | 0.841 |
| Czechia | 0.828 |
| Democratic People's Republic of Korea | 0.558 |
| Democratic Republic of the Congo | 0.382 |
| Denmark | 0.89 |
| Djibouti | 0.459 |
| Dominica | 0.729 |
| Dominican Republic | 0.592 |
| Ecuador | 0.64 |
| Egypt | 0.658 |
| El Salvador | 0.573 |
| Equatorial Guinea | 0.685 |
| Eritrea | 0.396 |
| Estonia | 0.835 |
| Eswatini | 0.577 |
| Ethiopia | 0.343 |
| Fiji | 0.664 |
| Finland | 0.856 |
| France | 0.834 |
| Gabon | 0.656 |
| Gambia | 0.399 |
| Georgia | 0.841 |
| Germany | 0.898 |
| Ghana | 0.557 |
| Greece | 0.794 |
| Greenland | 0.761 |
| Grenada | 0.669 |
| Guam | 0.813 |
| Guatemala | 0.526 |
| Guinea | 0.325 |
| Guinea-Bissau | 0.355 |
| Guyana | 0.618 |
| Haiti | 0.432 |
| Honduras | 0.496 |
| Hungary | 0.791 |
| Iceland | 0.869 |
| India | 0.566 |
| Indonesia | 0.66 |
| Iran | 0.67 |
| Iraq | 0.671 |
| Ireland | 0.867 |
| Israel | 0.803 |
| Italy | 0.801 |
| Jamaica | 0.684 |
| Japan | 0.87 |
| Jordan | 0.731 |
| Kazakhstan | 0.723 |
| Kenya | 0.508 |
| Kiribati | 0.527 |
| Kuwait | 0.851 |
| Kyrgyzstan | 0.596 |
| Laos | 0.49 |
| Latvia | 0.82 |
| Lebanon | 0.708 |
| Lesotho | 0.507 |
| Liberia | 0.37 |
| Libya | 0.709 |
| Lithuania | 0.843 |
| Luxembourg | 0.895 |
| Madagascar | 0.396 |
| Malawi | 0.384 |
| Malaysia | 0.737 |
| Maldives | 0.562 |
| Mali | 0.263 |
| Malta | 0.801 |
| Marshall Islands | 0.544 |
| Mauritania | 0.496 |
| Mauritius | 0.705 |
| Mexico | 0.649 |
| Micronesia (Federated States of) | 0.58 |
| Monaco | 0.902 |
| Mongolia | 0.606 |
| Montenegro | 0.791 |
| Morocco | 0.548 |
| Mozambique | 0.307 |
| Myanmar | 0.521 |
| Namibia | 0.612 |
| Nauru | 0.618 |
| Nepal | 0.422 |
| Netherlands | 0.883 |
| New Zealand | 0.84 |
| Nicaragua | 0.517 |
| Niger | 0.162 |
| Nigeria | 0.515 |
| Niue | 0.711 |
| North Macedonia | 0.744 |
| Northern Mariana Islands | 0.771 |
| Norway | 0.913 |
| Oman | 0.783 |
| Pakistan | 0.449 |
| Palau | 0.738 |
| Palestine | 0.588 |
| Panama | 0.686 |
| Papua New Guinea | 0.394 |
| Paraguay | 0.638 |
| Peru | 0.648 |
| Philippines | 0.623 |
| Poland | 0.802 |
| Portugal | 0.743 |
| Puerto Rico | 0.814 |
| Qatar | 0.83 |
| Republic of Korea | 0.878 |
| Republic of Moldova | 0.696 |
| Romania | 0.76 |
| Russia | 0.805 |
| Rwanda | 0.429 |
| Saint Kitts and Nevis | 0.746 |
| Saint Lucia | 0.67 |
| Saint Vincent and the Grenadines | 0.627 |
| Samoa | 0.641 |
| San Marino | 0.884 |
| São Tomé and PrÍncipe | 0.502 |
| Saudi Arabia | 0.805 |
| Senegal | 0.389 |
| Serbia | 0.767 |
| Seychelles | 0.724 |
| Sierra Leone | 0.347 |
| Singapore | 0.861 |
| Slovakia | 0.812 |
| Slovenia | 0.84 |
| Solomon Islands | 0.407 |
| Somalia | 0.081 |
| South Africa | 0.678 |
| South Sudan | 0.363 |
| Spain | 0.767 |
| Sri Lanka | 0.69 |
| Sudan | 0.515 |
| Suriname | 0.636 |
| Sweden | 0.872 |
| Switzerland | 0.929 |
| Syria | 0.619 |
| Taiwan (province of China) | 0.868 |
| Tajikistan | 0.539 |
| Thailand | 0.687 |
| Timor-Leste | 0.514 |
| Togo | 0.417 |
| Tokelau | 0.626 |
| Tonga | 0.636 |
| Trinidad and Tobago | 0.757 |
| Tunisia | 0.672 |
| Turkey | 0.748 |
| Turkmenistan | 0.67 |
| Tuvalu | 0.589 |
| Uganda | 0.404 |
| Ukraine | 0.736 |
| United Arab Emirates | 0.88 |
| United Kingdom | 0.847 |
| United Republic of Tanzania | 0.423 |
| United States of America | 0.859 |
| United States Virgin Islands | 0.799 |
| Uruguay | 0.697 |
| Uzbekistan | 0.631 |
| Vanuatu | 0.485 |
| Venezuela (Bolivarian Republic of) | 0.607 |
| Viet Nam | 0.617 |
| Yemen | 0.412 |
| Zambia | 0.505 |
| Zimbabwe | 0.476 |

Definition of SDI quintile: Low region (SDI index value range from 0 to 0.4547); Low-middle region (0.4547 to 0.6077); Middle region (0.6077 to 0.6895); High-middle region (0.6895 to 0.8051); High region (0.8051 to 1)
